# Computational Fluid Dynamics Analysis of the Fossil Crinoid *Encrinus liliiformis* (Echinodermata: Crinoidea)

**DOI:** 10.1371/journal.pone.0156408

**Published:** 2016-05-31

**Authors:** Janina F. Dynowski, James H. Nebelsick, Adrian Klein, Anita Roth-Nebelsick

**Affiliations:** 1 Staatliches Museum für Naturkunde Stuttgart, Stuttgart, Germany; 2 Fachbereich Geowissenschaften, Eberhard Karls Universität Tübingen, Tübingen, Germany; 3 Institut für Zoologie, Rheinische Friedrich-Wilhelms-Universität Bonn, Bonn, Germany; University of Lincoln, UNITED KINGDOM

## Abstract

Crinoids, members of the phylum Echinodermata, are passive suspension feeders and catch plankton without producing an active feeding current. Today, the stalked forms are known only from deep water habitats, where flow conditions are rather constant and feeding velocities relatively low. For feeding, they form a characteristic parabolic filtration fan with their arms recurved backwards into the current. The fossil record, in contrast, provides a large number of stalked crinoids that lived in shallow water settings, with more rapidly changing flow velocities and directions compared to the deep sea habitat of extant crinoids. In addition, some of the fossil representatives were possibly not as flexible as today’s crinoids and for those forms alternative feeding positions were assumed. One of these fossil crinoids is *Encrinus liliiformis*, which lived during the middle Triassic Muschelkalk in Central Europe. The presented project investigates different feeding postures using Computational Fluid Dynamics to analyze flow patterns forming around the crown of *E*. *liliiformis*, including experimental validation by Particle Image Velocimetry. The study comprises the analysis of different flow directions, velocities, as well as crown orientations. Results show that inflow from lateral and oral leads to direct transport of plankton particles into the crown and onto the oral surface. With current coming from the “rear” (aboral) side of the crinoid, the conical opening of the crown produces a backward oriented flow in its wake that transports particles into the crown. The results suggest that a conical feeding position may have been less dependent on stable flow conditions compared to the parabolic filtration fan. It is thus assumed that the conical feeding posture of *E*. *liliiformis* was suitable for feeding under dynamically changing flow conditions typical for the shallow marine setting of the Upper Muschelkalk.

## Introduction

**Fig 1:**
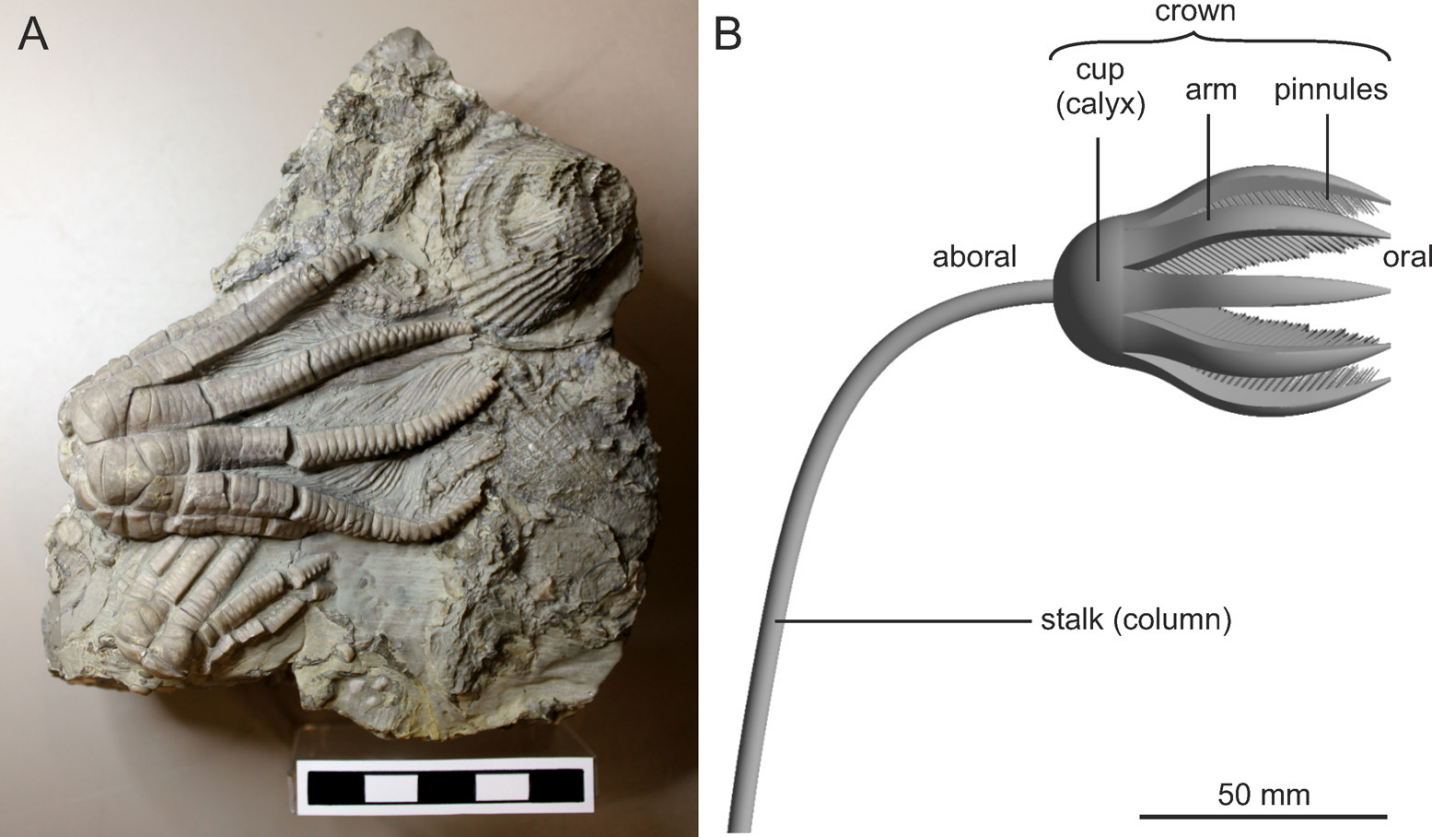
*Encrinus liliiformis*. (A) Fossil crown from Crailsheim, Germany, showing typical preservation with arms opened to a slight extent (deposited in the collection of the State Museum of Natural History Stuttgart, specimen number SMNS21859). (B) Schematic illustration of morphological features.

Crinoids today live in all oceans, from littoral settings to about 9000 m water depth [1]. In general, two main groups are recognized: the stalked sea lilies, which today are restricted to deep sea environments, and the unstalked feather stars, which occur in various environments, from the deep sea to shallow water habitats [2]. Stalked crinoids have existed since the early Palaeozoic and the fossil representatives, in contrast to living ones, were highly abundant and diverse in shallow water settings. Members of the class Crinoidea share a similar basic shape with the major organs located in the small cup or calyx, and a skeleton that is constructed of ossicles of high Mg calcite, which is covered by a thin integument [3] (Fig 1, S1 Fig). The arms, making up the filter apparatus, possess numerous pinnules, which in turn bear hundreds of small ambulacral tube feet, to which nutritive particles adhere [4–7].

Crinoids are passive suspension feeding animals thus depending on the movement of the surrounding water mass to supply them with food [8, 9]. Their main diet consists of miscellaneous planktonic organisms, with diameters typically less than 200 μm [10, 11]. Living stalked crinoids, which have only limited ability for locomotion [12], show a typical feeding position, with the arms bent into the incoming water flow, thus creating a simple but effective three dimensional filter, the so called parabolic filtration fan [13] (S1 Fig). In this posture, the concave side of the filter apparatus faces the current directly, while the particle catching tube feet are located on the leeward side. This leeward feeding with a dish-shaped filter is known from various suspension feeders and is interpreted as to increase fluid flux through the filter and particle capture efficiency in comparison to a planar arrangement [8, 14]. On the leeward side of such a filter, the velocity is reduced and turbulence occurs that leads to a recirculation of particles onto the filtering surface [8]. In crinoids, this feeding position is confined to special flow conditions, including unidirectional flow with velocities of up to 0.25 m/s [13,15]. Below a special threshold (approximately 0.01 m/s), insufficient numbers of particles would reach the animal to compensate the energy needed for capture, mainly due to the influence of the boundary layer around the feeding structures preventing plankton to penetrate through it and touch the sticky surface [16–18]. In slack currents, crinoids typically adopt an upright position with the oral surface facing upwards, resembling a wilted flower [13, 19, 20]. In high flow velocities, the drag on the crown and thus the strain on the stalk increases to such an extent that the crinoid is in danger of becoming detached from the ocean floor once the force reaches a critical value. In strong currents, stalked crinoids close their arms forming a drag reducing tear shape, called the survival posture [21, 22] to withstand periods of stronger flow without being injured. In this position, no filtering activity is possible.

Fossil crinoids show a great diversity in morphology with many possessing special morphologic features, such as sensory organs in camerate crinoids [21] or the particular wing plates of the camerate crinoid *Pterotocrinus* [23, 24]. Almost all hydrodynamic studies thus far on fossil crinoids have focused on Palaeozoic forms analyzing species that were obviously similar to extant taxa with respect to bending their arms into the parabolic filtration fan [21–27]. Experimental studies were performed with models of *Pterotocrinus* forming a feeding posture similar to the parabolic filtration fan [23]. Varying orientation in the flow showed that a leeward position of the filtering surface resulted in decreased velocities and eddies in the wake of the crown, which probably supported the successful capture of nutritive particles.

Not all of the extinct representatives, however, were able to use the parabolic filtration fan to capture plankton out of the water. The Palaeozoic *Ammonicrinus* is reconstructed as a soft-bottom dweller, and instead of being lifted into the typical upright position of most stalked crinoids, the body was lying on the sea floor [28]. Some fossil crinoids had short and quite stout arms which may have been less flexible than in extant taxa, and for which crown postures other than the parabolic filtration were assumed (e.g. [29]). One of these examples is the Middle Triassic *Encrinus liliiformis*, which serves as a model crinoid in the presented study. Contrary to the reconstruction as an active filter feeder [30], *E*. *liliiformis* herein is supposed to have used passive suspension feeding as its main food gathering strategy, similar to that of all living crinoids.

Based on the work of [31], several studies have investigated the mechanisms of particle capture that are involved in suspension feeding [16, 32–37]. Following these studies, two encounter mechanisms are of high importance: 1) direct interception, and 2) inertial impaction. The first mechanism results in capture when a particle follows a streamline, which comes within a distance of one particle radius to the filtering element. The second mechanism involves the inertia of the particle, where the density difference compared to the fluid causes a deviation of the particle from the streamline such that the particle passes within one particle radius and impacts the filtering structure [31]. All mechanisms of successful particle capture belong–since occurring close to a solid surface—to the realm of low to intermediate Reynolds numbers.

Finally, this part of the filter feeding process is for larger filter systems, such as the crinoid crown, based upon a sufficient transport of particles to the vicinity of the ultimate filtering elements, and therefore upon the flow pattern around the crown. This flow process represents the regime of higher Reynolds numbers (referred to as “macroscopic flow” throughout the rest of the text). This is particularly valid to filtering organisms such as stalked crinoids in which the filtering elements are attached to a large structure (here the crown formed by the arms) and are elevated to reach currents of higher flow velocity. While general particle capture mechanisms of passive suspension feeders have been studied in detail, the flow pattern around crinoids, especially of those differing in morphology from extant taxa, has been neglected widely (see [22, 23] for general observations and theoretical considerations). For the reconstruction of palaeoenvironments, however, as well as for conclusions concerning particle encounter, the feeding position and thus ambient flow regime is of high importance [34, 35]. The presented study complements the current knowledge on particle capture mechanisms involved in crinoid feeding by examining the macroscopic flow patterns forming around the crown of *E*. *liliiformis*.

The aim of the present study is to evaluate the flow pattern around a fossil crinoid crown adopting a feeding position different to the parabolic filtration fan of extant crinoids. In this different posture, the calyx and arms form a tear shape instead of backward bending of arms, as suggested by various authors [30, 38, 39], and the question is addressed whether or not the flow pattern developing around such an object allows for particle transport into the filter apparatus. The underlying rationale is that various fossil crinoid taxa probably lived under more dynamic conditions than extant crinoids, in shallow water and under higher current velocities. The functionality of the tear-shaped feeding posture suggested for *E*. *liliiformis* depends to a large degree on the macroscopic flow pattern around the crown which dictates the transport of nutrients into the vicinity of the filter elements. To this end, flow around fossil crinoids was simulated for different flow velocities as well as flow directions. In addition, effects of variations in the position of different body parts as well as the behaviour of different sized plankton particles were analyzed.

## Materials and Methods

### Encrinus liliiformis

The analyzed crinoid *Encrinus liliiformis* Lamarck, 1801, a member of the subclass Articulata, order Encrinida, lived during the Anisian to lower Carnian in Central Europe [40]. It had 10 short, biserial arms, a low bowl shaped cup and a long stalk consisting of cylindrical columnals. The palaeontological collection of the State Museum of Natural History Stuttgart (SMNS) houses a large number of *E*. *liliiformis* specimens from southwest Germany, so that no further field work was necessary for the presented study. Specimen number SMNS21859 (Fig 1A), originating from Crailsheim, Baden-Württemberg, shows the typical preservation of these crinoids, with a slight opening of the arms and exposed pinnules, and served as an example to build the models analyzed herein.

As typical for all echinoderms, sea lilies have the ability to actively shed parts of their body and regenerate them, a process known as autotomy. Crinoids typically autotomize parts of the stalk or arms and many fossils of *E*. *liliiformis* are known, where parts of the arms are missing, which might be attributed to a stress reaction during the dying process. Others have regenerated arms or sealed stem ruptures, where the loss could also have been a result of non-lethal predatory attack [38].

Following the observations and reconstructions of [30], [38] and [39], it was suggested that the arms of *E*. *liliiformis* were not bent backwards to form the parabolic filtration fan during feeding as can be observed in living stalked crinoids, and an alternative feeding position was assumed instead (Fig 1B): the arms formed a tear shape and were held at an angle of about 45° in relation to the long axis. Recent crinoids open their filtration fan by contraction of aboral ligaments, while the inward bending is caused by contraction of oral muscles [41]. From morphology alone it is hard to tell if *E*. *liliiformis* was able to form the parabolic filtration or how wide the arms could open [30, 38], but the rather stout arms as well as the typical preservation posture of numerous specimens indicate a differing feeding position. The stalk of *E*. *liliiformis* lacks muscular articulations, and was only flexible in the most proximal part where the columnals show not only shorter, but also varying diameters compared to the rest of the stalk. This is interpreted to allow for the crown to be oriented parallel to the direction of flow. A rapid active movement of the crown, as would be necessary to react to changing flow directions typical of shallow water conditions, however, was not possible.

**Fig 2:**
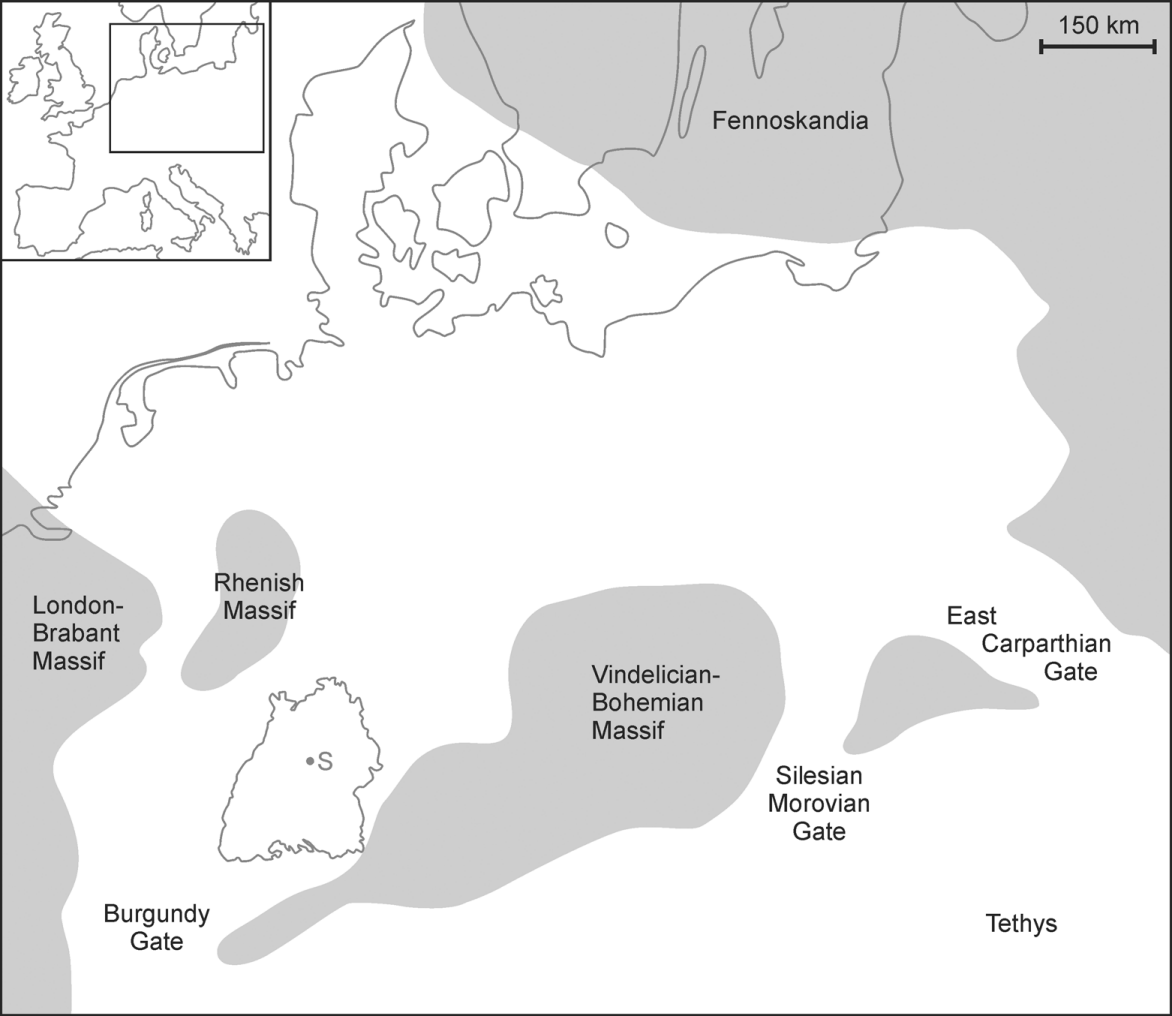
Palaeogeography of the Central European Basin during the upper Muschelkalk (Middle Triassic). Grey colour: terrestrial settings, white colour: marine settings, outline displays state of Baden-Württemberg with S: Stuttgart, Germany. Modified following [38, 43].

In the Triassic period, Germany was part of the Central European Basin [42] and during the middle Triassic Muschelkalk (Anisian to early Ladinian), marine carbonates were deposited due to a transgression of the Tethys Ocean. In Southern Germany (Fig 2), fossiliferous sediments formed in a restricted part of this peripheral sea [38, 43]. The region around Crailsheim, about 80 km to the north-east of Stuttgart, is famous for mass accumulations of crinoid stem fragments, the so called ‘Trochitenkalk’ of the Crailsheim member. These sediments, which reach a thickness of up to 16 m, formed under shallow marine conditions (above storm wave base respectively in parts also above fair weather wave base) on a gently inclined carbonate ramp [38, 44–47].

This habitat of *E*. *liliiformis* is completely different to that of extant stalked crinoids living in deep sea environments where changes of flow conditions only occur gradually and unidirectional flow prevails over longer time periods. In shallow water habitats, as represented by the marine setting of the Triassic, in which *E*. *liliiformis* lived, flow conditions can vary within short time intervals, with higher velocities and more rapidly changing directions. These subtidal settings were still influenced by wave action, but not as intensely as in wave swept environments with oscillating flow. Specimens originating from Neckarwestheim, about 70 km to the west of Crailsheim, however, were found in sediments which formed in a deeper part of the basin under more stable conditions and longer periods of unidirectional flow. These sediments comprise intercalations of marls and clays and were deposited between wave base and storm wave base [38], with an assumed water depth of no more than 180 m [39, 48]. Specimens from Neckarwestheim differ from those of Crailsheim in several morphological characters, including longer arms with a more pronounced ornamentation on the outer surface [49].

### Computational Fluid Dynamics

**Fig 3:**
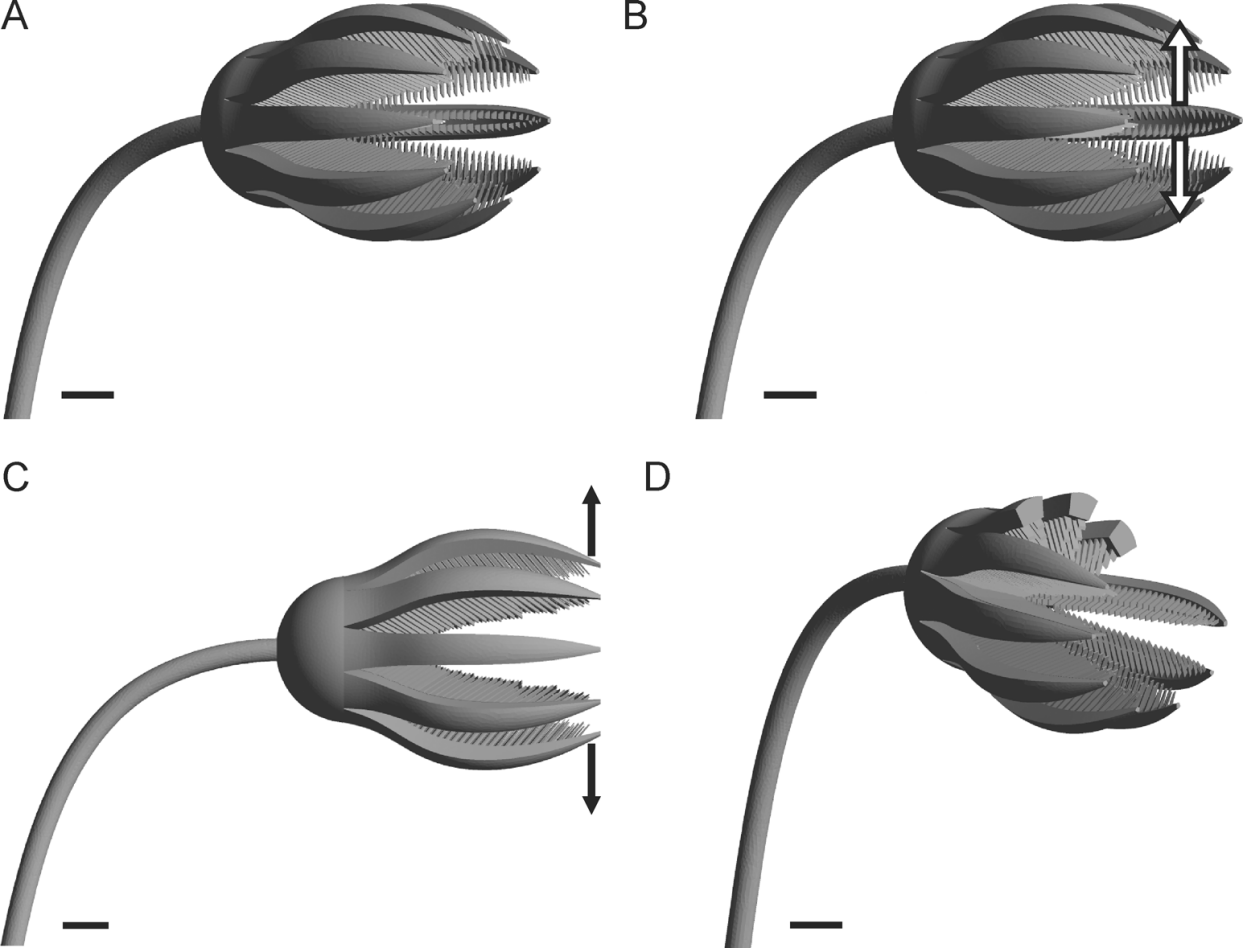
Analyzed 3D models of *E*. *liliiformis*. (A) Base model. (B) Pinnules spread. (C) Arms opened. (D) Parts of 3 arms capped.

Fluid flow around computer generated 3D models of *E*. *liliiformis* (Fig 3) was calculated by applying Computational Fluid Dynamics (CFD). This method is based on the numerical solution of partial differential equations, according to the defined boundary conditions of the analyzed computational regime. The presented study used the finite volume based software package CFX of the ANSYS® Academic Research, Release 13 (for solving and post-processing) and Release 15 (for post-processing) as well as the software Intelligent Light FIELDVIEW 15 (for post-processing). In total, 4 different geometries, consisting of the skeletal body parts (stalk, calyx, arms and pinnules) with dimensions representing an average morphology, following values provided by [49], were created: model 1 represents the base geometry with a moderate opening angle of the arms (Fig 3A); in model 2 the arms take in the same position as in model 1, but the pinnules are spread laterally (Fig 3B); model 3 shows an opening of the arms by 10 mm, corresponding to an increase in the diameter of the crown by 20 mm (Fig 3C); and model 4 reflects loss of parts of the arms by capping the distal portion of 3 arms (Fig 3D). The crinoid models were surrounded by a fluid domain of a cuboidal rectangular shape, showing a length of 0.7 m, and a width and height of 0.28 m. The distance between the inlet and the crinoid model was about 3 times the crown length, and between the model and the outlet 5 times the crown length. Sensitivity analyses, based on simulations using larger computational domains, including larger distances of inlet and outlet as well as between the walls and the crown, did not result in considerable changes of the observed flow patterns.

**Fig 4:**
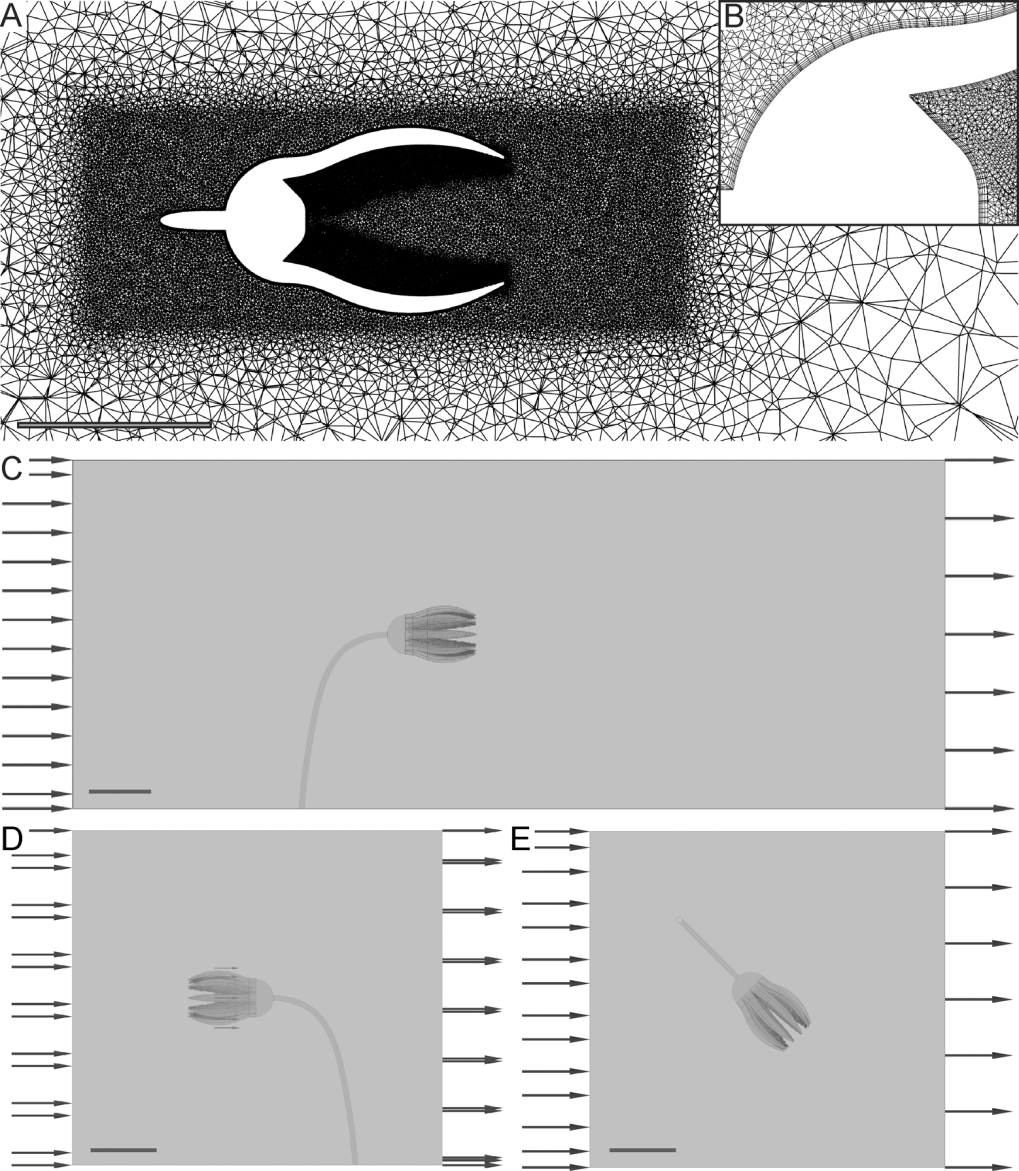
CFD setup. (A) Overview of computational mesh of the base model. (B) Detail of (A) illustrating inflation layers on the model surface. (C) Computational domain with inflow from aboral. (D) Computational domain with inflow from oral. (E) Computational domain with inflow from lateral.

Resulting meshes are built up of tetrahedral elements with a refinement area surrounding the crown including inflation layers (Fig 4A and 4B), and element numbers ranging between 6,261,045 and 8,298,277. Inflation layers consist of prisms forming a structured grid thus enabling a better numerical basis for the calculation of the boundary layer. Mesh refinement studies showed good convergence and an increase in element number did not result in considerable changes of velocities. In the present study, the boundary conditions of the setup include a fluid domain using ocean water parameters (average density = 1026.021 kg/m^3^, dynamic viscosity = 0.00122 Pa·s), with unidirectional flow (parallel to the x-axis, velocity vector u/v/w = 1/0/0) from the aboral (Fig 4C), oral (Fig 4D) and lateral (Fig 4E) side of the crinoid. The boundaries were set as: 1) an inlet boundary with initial velocities (Vel_init_) of 0.03 m/s, 0.14 m/s and 0.5 m/s respectively, and medium intensity turbulence (= 5%); 2) an outlet boundary with 0 Pa average static pressure; 3) no slip wall conditions for the crinoid model; and 4) free slip wall conditions for the 4 walls representing the fluid to best approximate an open water domain. Using crown length parallel to the inflow direction as characteristic length, the Reynolds numbers for all analyzed scenarios take values between 1,317 and 30,729 thus indicating a turbulent global flow regime.

Steady state simulations were carried out with all geometries, based on the standard k-epsilon-turbulence model (a two-equation RANS model) with scalable wall functions (see ANSYS® Academic Research, Release 13, Help System, ANSYS CFX-Solver Modeling Guide, ANSYS, Inc.). To reproduce the behaviour of potential plankton in the flow, Lagrangian particle tracking simulations were conducted with a particle density of rho = 1080 kg/m^3^ (c.f. [50]) and diameters ranging between 10 and 500 μm. A total number of 1000 particles was introduced, distributed randomly over a planar, circular plane with a radius of 25 mm for aboral and oral inflow, and 40 mm for lateral inflow, positioned in front of the crinoid with a distance of 100 mm to the crown. The size of the “particle plane” was selected following preliminary studies, which allowed for identifying the required area that had to be covered for particle injection. The same particle start positions were used for all analyzed geometries. To evaluate the effect of starting particle number, simulations introducing 1000, 2000 and 3000 particles were performed for the base model and the one with arms opened for an aboral inflow and for a velocity of 0.14 m/s (Table 1). Absolute percentages of particles that are redirected into the crown vary with particle number for both models, with differences up to 25% between 1000 and 3000 particles for the base model and 5% between 1000 and 3000 particles for the model with arms opened. In the following, a number of 1000 particles was selected as base value. The solving process was performed using computer resources of the bwGRiD project (http://www.bw-grid.de) and the bwUniCluster (http://www.bwhpc-c5.de). Simulations were run on four cores, and needed a mean wall clock time of 1 hour to converge (standard convergence criteria set to RMS values lower than 1.0E-4) which in most simulations was reached in less than 30 iterations (none needed more than 50 iterations). Velocity distributions are illustrated as contour, linegraph and isosurface plots, flow directions as vector plots, and particle motions as pathline plots.

**Table 1:** Absolute percentages of particles entering the crown (aboral inflow) to evaluate the effect of changing starting particle number.

Geometry	1000 particles	2000 particles	3000 particles
Base model	1.5%	1.7%	2.0%
Arms opened	3.4%	3.2%	3.2%

### Particle Image Velocimetry

**Fig 5:**
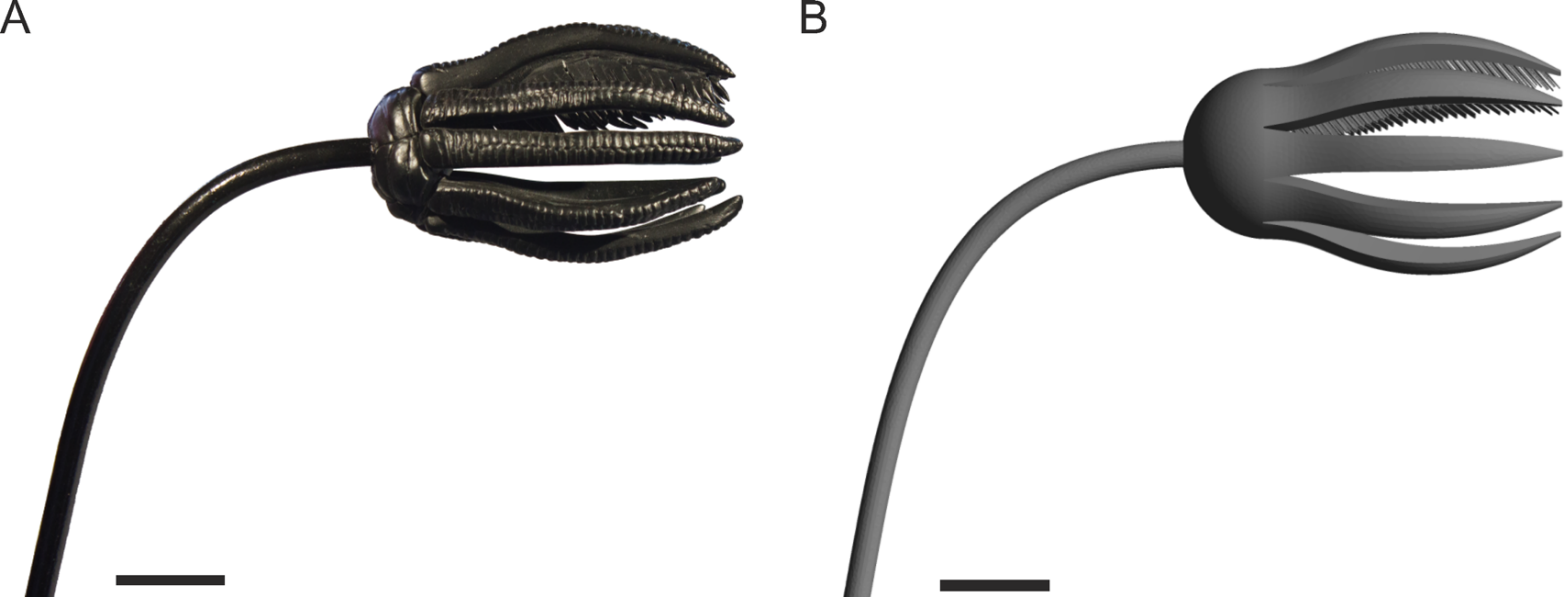
Models used for validation of CFD simulations. (A) Handmade resin-wire model. (B) computer-generated 3D model. Scale bars 20 mm.

For general validation of CFD results, a simplified handmade model of the base geometry (Fig 5A) was analyzed in a recirculating flow tank applying 2D Particle Image Velocimetry (PIV) at the Zoological Institute of the University of Bonn. For the experiments, a water resistant model of *E*. *liliiformis* was constructed using a combination of epoxy resin (Apoxie®Sculpt), wire and flexible tubes, painted with black matt car finish. The morphology was modelled down to the skeletal elements represented by the arms and pinnules (with three instead of all ten arms covered with pinnules). The model was analyzed in a fresh water flow tank, with incoming flow from the aboral side, in three different bulk flow velocities (Vel_init_ = 0.14 m/s, 0.166 m/s and 0.213 m/s).

PIV is a laser optical method used to visualize flow profiles around objects. In the present setup, a laser sheet (Pegasus, 527 nm) was adjusted in the X-Z-plane of the flow tank (measurement area size 134.7401x134.7401 mm). The origin of Y axis was defined as the centre of the crinoid crown. Seeding particles (PSP 50, Dantec Dynamics) were illuminated with laser light. A high-speed video camera (Photron Ultima APX) was adjusted orthogonal to the light plane underneath the flow tank and filmed illuminated particles with 250 frames per second (FPS). PIV was done for 5 light sheet levels adjusted at Y = -4, -2, 0, 2 and 4 cm. Flow vectors were computed with DaVis (LaVision). Image calibration was done using a two level calibration plate (Typ 20, LaVision).

The PIV measurements provided values of the two velocity components V (longitudinal flow, parallel to the x-axis = streamwise) and U (perpendicular to the incoming flow direction, parallel to the z-axis = crosswise) measured at 128x128 sampling points distributed on a regular grid across the measurement area. The values at light-sheet level Y = 0 (centre of the crown) yielded the best results for the interpretation of flow patterns. For evaluating results of CFD, simulations were set up copying the conditions of the flow tank experiments, including dimensions of the tank, morphology and orientation of the model (Fig 5B), fresh water as fluid and the three bulk flow velocities.

### Validation

**Fig 6:**
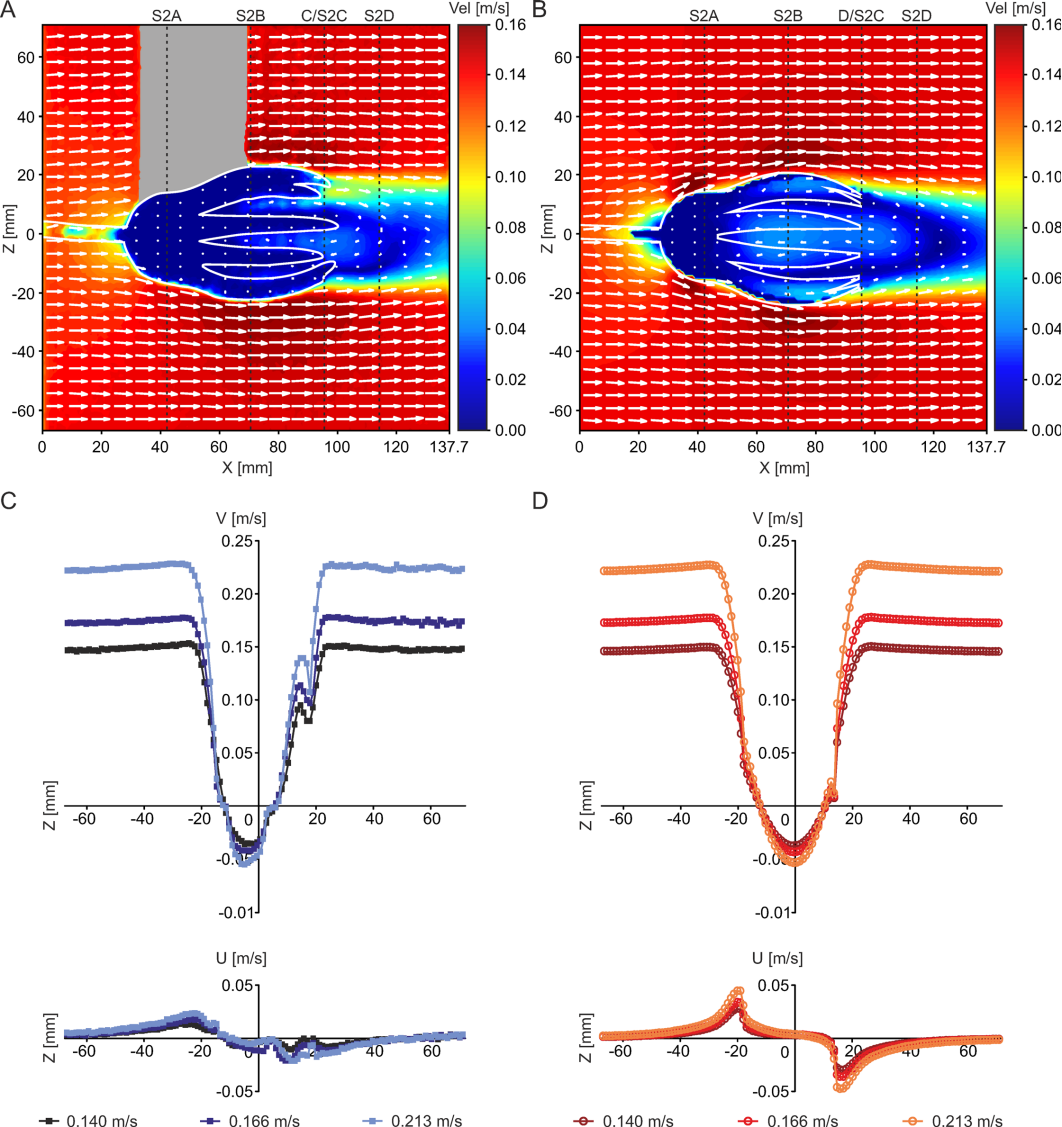
Summarized results of PIV experiments and CFD simulations. (A) Combined contour-vector plot illustrating PIV results at V_init_ = 0.14 m/s. (B) Combined contour-vector plot illustrating CFD results at V_init_ = 0.14 m/s. (C) Velocity component V and U of PIV experiments for all three inflow velocities, measured along a line parallel to the z-axis (location indicated by dotted line in (A)). (D) Velocity component V and U of CFD simulations for all three inflow velocities, measured along a line parallel to the z-axis (location indicated by dotted line in (B)).

The comparison of CFD and PIV results generally show good agreement as a recirculation area develops behind the crown with both methods and under all analyzed inflow velocities. The combined contour-vector plots (Fig 6A and 6B) illustrate similar velocity distributions as well as flow directions for experimental and computational results. The comparison of velocity component V and U (Fig 6C and 6D), measured along a line parallel to the z-axis directly behind the end of the arms (location indicated in Fig 6A and 6B), shows similar extent as well as strength of the recirculation zone. An increase in inflow velocity does not change the general flow pattern, and shows the same trends in PIV as well as CFD. Slight differences between PIV and CFD results can be attributed to irregularities of the handmade model compared to the perfectly smooth and symmetrical computer generated geometry. More detailed information is provided in S2 Fig, and [51].

## Results

### Aboral inflow

**Fig 7:**
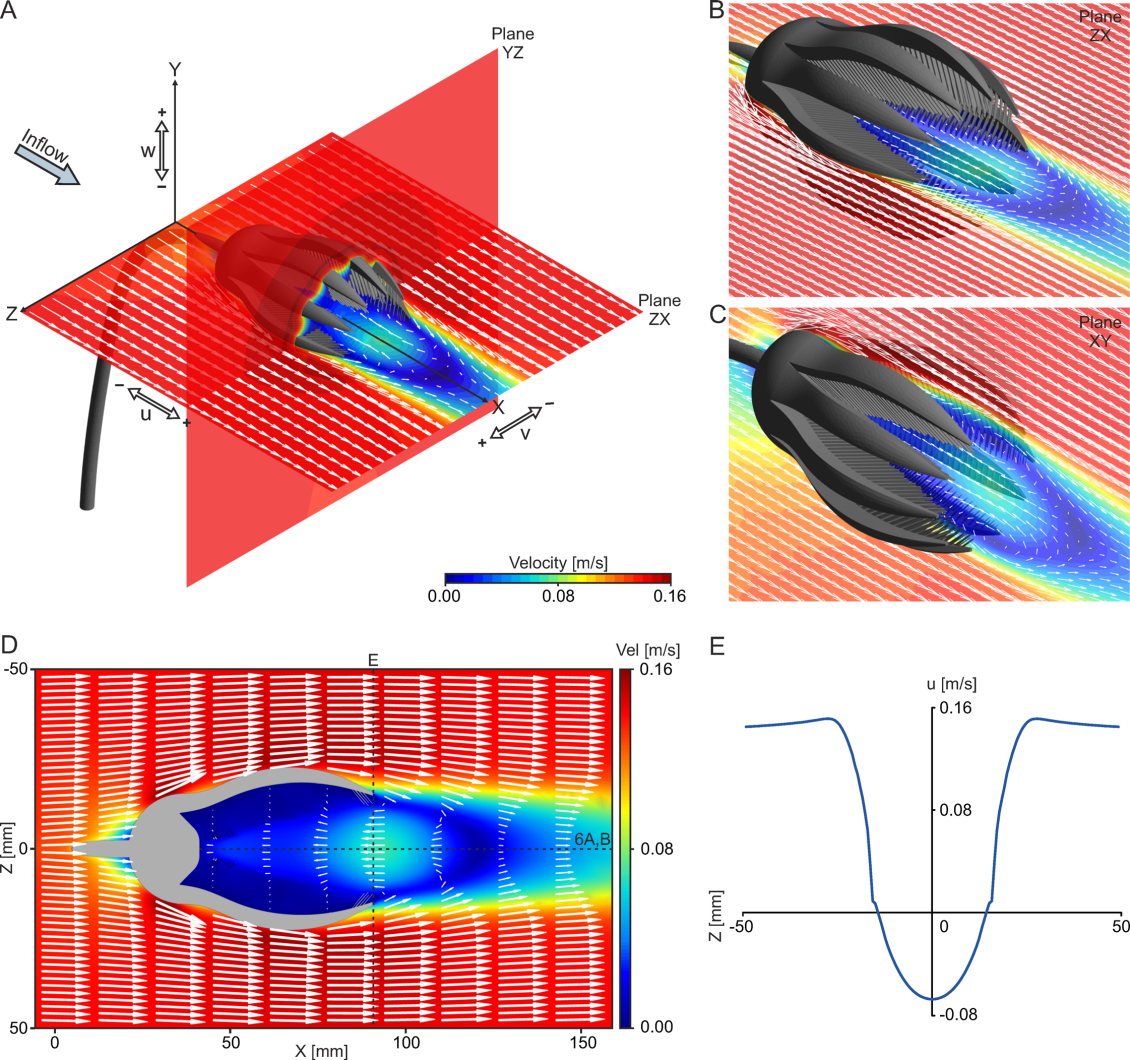
Flow results for the base model and aboral inflow at V_init_ = 0.14 m/s. (A) Overview illustrating model orientation and individual velocity components. (B) Combined contour-vector plot illustrating recirculation on ZX plane in oblique view. (C) Combined contour-vector plot illustrating recirculation on XY plane in oblique view. (D) Combined contour-vector plot illustrating recirculation on ZX plane in top view. (E) Linegraph plot of velocity component u directly behind the end of the arms (location indicated by dotted line in (D)). Negative u values indicate recirculation zone.

This setting corresponds to Fig 4C. The tear-shaped crown creates a recirculation zone developing right behind the end of the arms (Fig 7). For all of the three analyzed inflow velocities and all of the four models, this zone leads to a backward oriented transport of water and particles into the crown. The general flow pattern, illustrated here for the base model at V_init_ = 0.14 m/s as combined contour-vector plots (Fig 7A–7D), indicates an increase in velocity when the flow passes the exterior of the crown (darker red colours of the contour plots), caused by the curved surface, at the maximum diameter of the calyx and the widest opening of the arms. In the wake of the crown, a decrease in velocity can be observed (darker blue colours). The vectors indicate a reversal of flow direction, with recirculation starting at the tip of the arms, where the flow is redirected into the filter apparatus. In the centre of the recirculation area, the velocity again increases (lighter blue colours). Reverse flow, in relation to the inflow direction, is represented by negative values of velocity component u (Fig 7E), and is here denoted as u_recirc_. For the base model, the strongest reverse flow (measured along the x-axis) reaches values of u_recirc_ = -0.0681 m/s, and thus 48.63% of the inlet velocity (V_init_ = 0.14 m/s). In comparison to the base model, an opening of the arms increases u_recirc_ for all inflow velocities, while the lowest recirculation velocities result with parts of 3 arms capped. At V_init_ = 0.03 m/s and 0.14 m/s, spreading of the pinnules has only little effect on u_recirc_, while it results in considerably weaker reverse flow velocities at V_init_ = 0.50 m/s. Values of u_recirc_ represented in % of V_init_ are given in Table 2, for all models and inflow velocities.

**Table 2:** Values of u_recirc_ in % of V_init_, measured along x-axis, and number of particles transported backwards into the crown (in square brackets) for all 3D models and inflow velocities.

V_init_ [m/s]	Base model	Pinnules spread	Arms opened	3 arms capped
0.03	23.70% [5]	24.39% [6]	26.12% [13]	22.06% [5]
0.14	48.63% [15]	47.45% [16]	52.07% [34]	42.68% [11]
0.50	63.72% [17]	53.80% [10]	71.19% [27]	50.94% [13]

**Fig 8:**
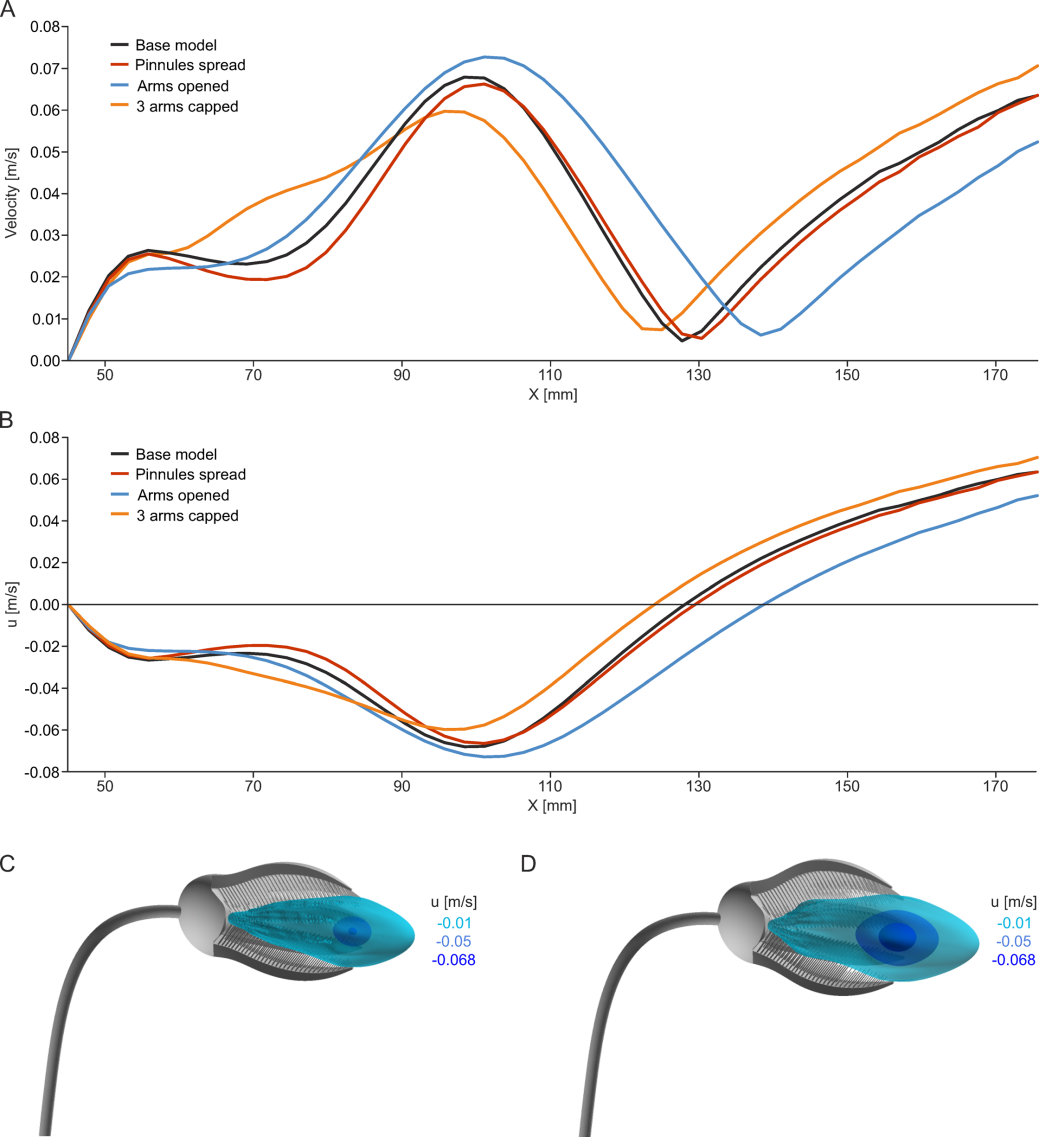
Results of the four different models for aboral inflow at V_init_ = 0.14 m/s. (A) Linegraph plots of total velocity parallel to the x-axis, starting at the inner surface of the calyx (location indicated by dotted line in Fig 7D). (B) Linegraph plots of velocity component u parallel to the x-axis, starting at the inner surface of the calyx (location indicated by dotted line in Fig 7D). (C) Isosurface plots of negative u values for the base model. (D) Isosurface plots of negative u values for the model with arms opened.

The linegraph plots of total velocity and velocity component u along the x-axis (Fig 8A and 8B) show that spreading of pinnules has a low effect on the velocity distribution of the base model, whereas the opening of the arms not only leads to an increase in u_recirc_, but additionally to an enlargement of the recirculation zone in x-direction (S3 Fig). Loss of parts of arms has the opposite effect and results in somewhat higher velocities inside the crown (between x = 60–85 mm). The isosurface plots of negative u-values illustrate the 3dimensional extent of the recirculation zone, reaching well into the inside of the crown in both the base model (Fig 8C) and the one with arms opened (Fig 8D). Due to the opening of the arms, the recirculation zone enlarges in all three dimensions and the increase in u_recirc_ is indicated by the expansion of the dark blue isosurfaces (compared to the base model).

**Fig 9:**
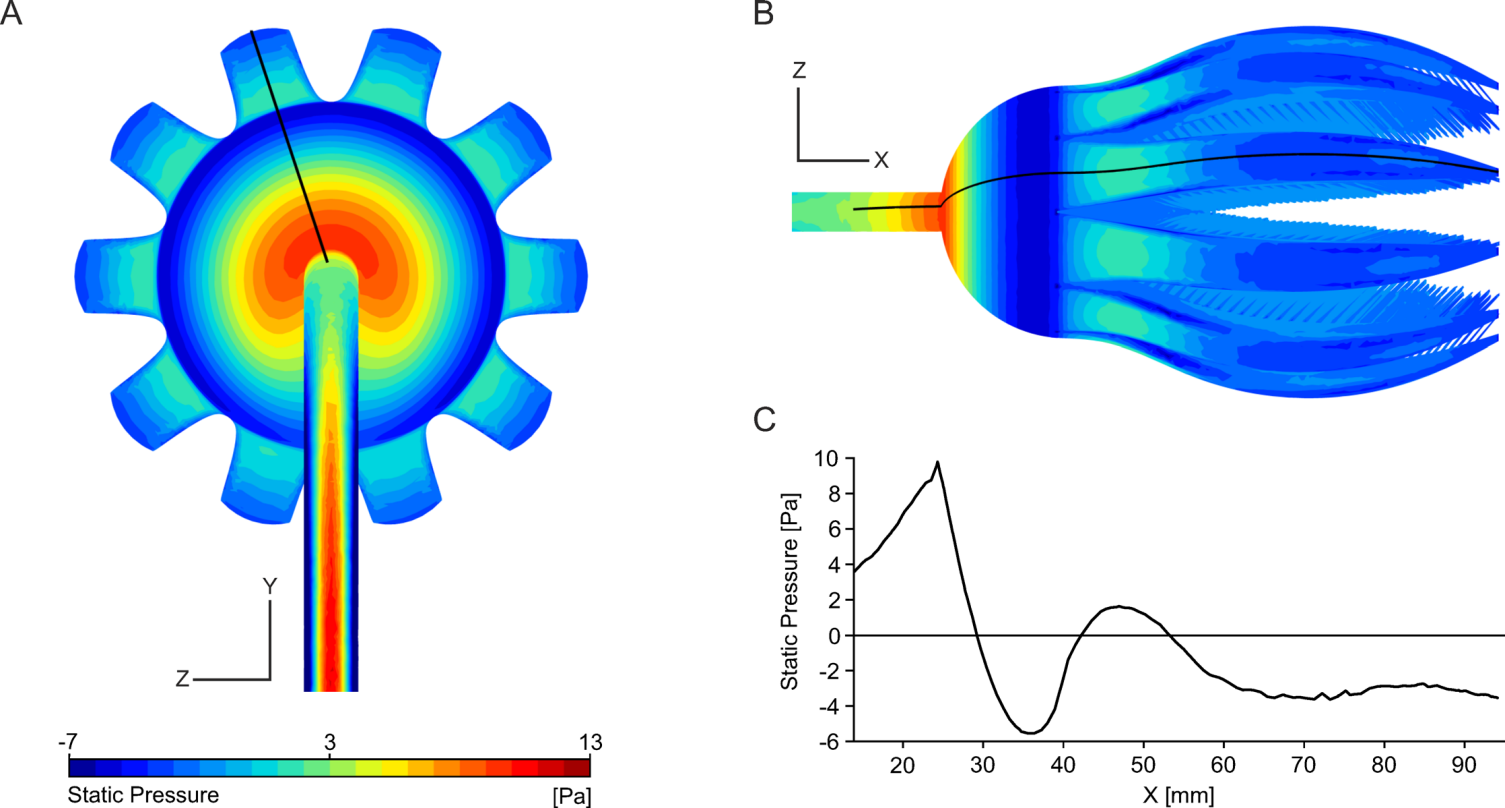
Pressure distribution on the base model at V_init_ = 0.14 m/s. (A) Contour plot of p_stat_ on the aboral surface of the base model. (B) Contour plot of pstat in top view. (C) Linegraph plot of p_stat_ plotted against x, following the outline of the crinoid model (location indicated in (B)).

Since the k-epsilon turbulence model does not simulate the flow processes within the boundary layer but computes (via wall functions) an approximation of the near-wall region, values for this area such as pressure as well as drag are also necessarily approximate. Despite this circumstance, the pressure distribution over the model will be shown in the following with the caveat that these values should be understood as rough values. Drag values of the CFD simulations are provided with the same caveat in S1 Table. The contour plots of the static pressure p_stat_ (Fig 9A and 9B), given in relation to the ambient pressure, show that the highest pressure peaks on the crown occur at the calyx, with p_stat_ = 9.77 Pa. Following the outline of the crinoid model (Fig 9C), minimum values are reached at the widest calyx diameter with p_stat_ = -5.55 Pa, then the pressure increases again to positive values of p_stat_ = 1.62 Pa where the arms curve outwards, and finally decreases constantly following the arm with negative p_stat_ until the tip of the arm is reached.

**Fig 10:**
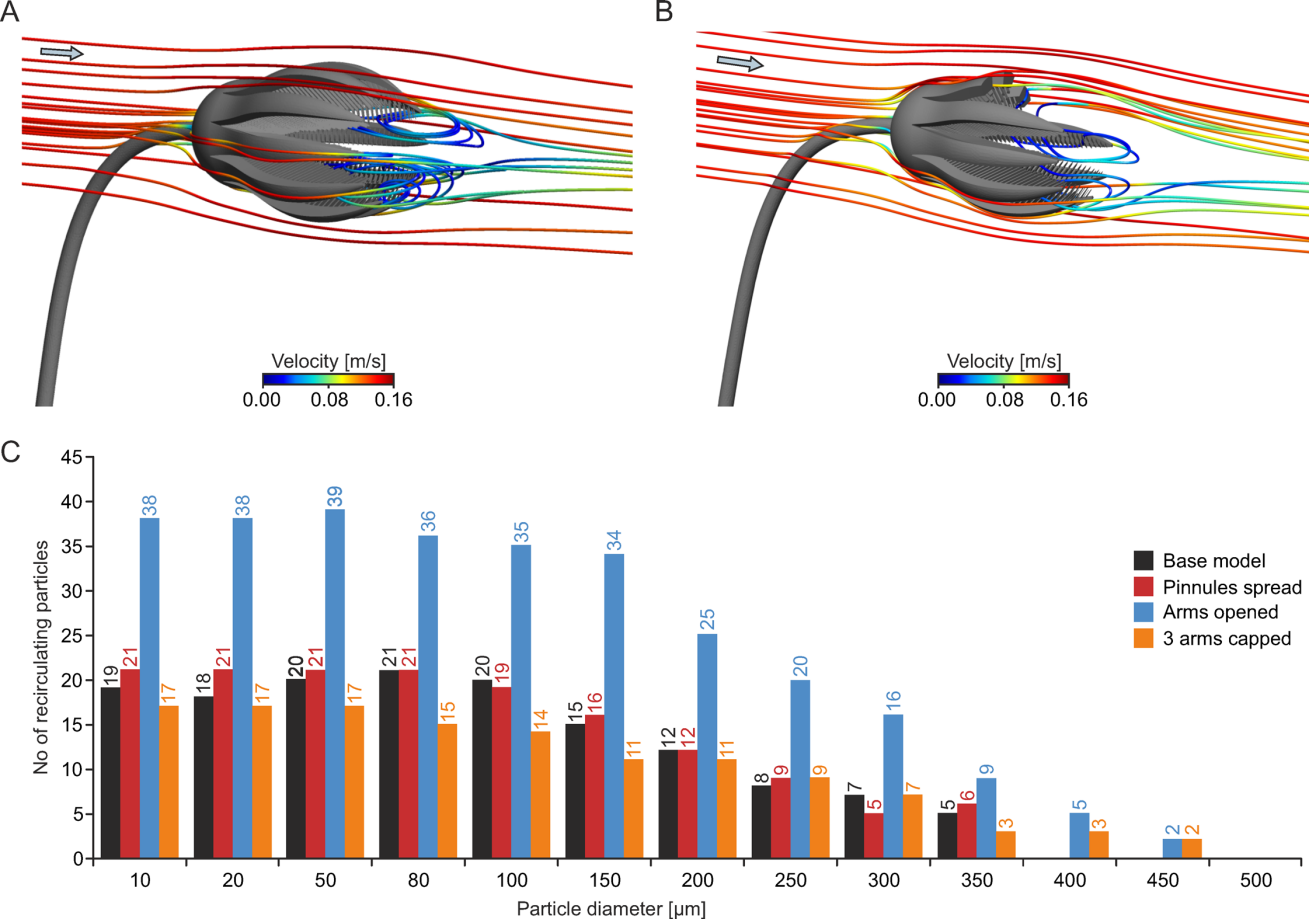
Results of particle tracking simulations for aboral inflow at V_init_ = 0.14 m/s. (A) Pathline plot illustrating recirculation of plankton particles (diameter = 150 μm) into the filter apparatus for the base model. (B) Pathline plot illustrating recirculation of plankton particles (diameter = 150 μm) into the filter apparatus for the model with 3 arms capped. (C) Histogram showing number of recirculating particles with different diameters for all four models.

Results of particle tracking reveal that potential plankton particles can be caught in the recirculation current, and are transported back onto the filtering surface of the crinoid and pass through the pinnules (Fig 10A and 10B, S1 and S2 Videos). Those particles that experience negative values of velocity component u and which are therefore transported backwards into the crown are denoted in the following as recirculating particles. Again, spreading of the pinnules has only little effect compared to the base model (Table 2). Opening of the arms, however, increases the number of recirculating particles considerably (Table 2 and Fig 10C). In addition, the number of loops the particles perform inside the crown rises thus enhancing the capture probability. All model variations show that increasing flow velocity does not lead to a parallel increase of particles that are transported into the crown. Beyond 0.14 m/s, the number or redirected particles remains almost constant, despite a higher recirculation velocity (Table 2). The model with arms opened shows highest transport of particles into the crown, because its recirculation zone is largest (Fig 8). This means that the rate of particles transported backwards into the crown depends on arm position and is to a large degree independent of flow velocity.

When parts of three arms are capped, recirculation of plankton still occurs, but the number of particles decreases, and most of these are transported through the gap in the upper part of the crown without approaching the filtering surface (Fig 10B, S3 Video). For the base model and with pinnules spread, highest numbers of recirculating particles are reached for particle diameters smaller than 100 μm (Fig 10C), and particles larger than 350 μm are not transported backwards. With arms opened, the diameter preference rises to 150 μm, and the maximum diameter of particles that recirculate, to 450 μm. When three arms are capped, the number of recirculating particles reaches slightly lower values compared to the base model, but as already mentioned above, many of these are transported through the gap in the crown. Similar to the model with arms opened, particles of up to 450 μm recirculate.

### Oral inflow

**Fig 11:**
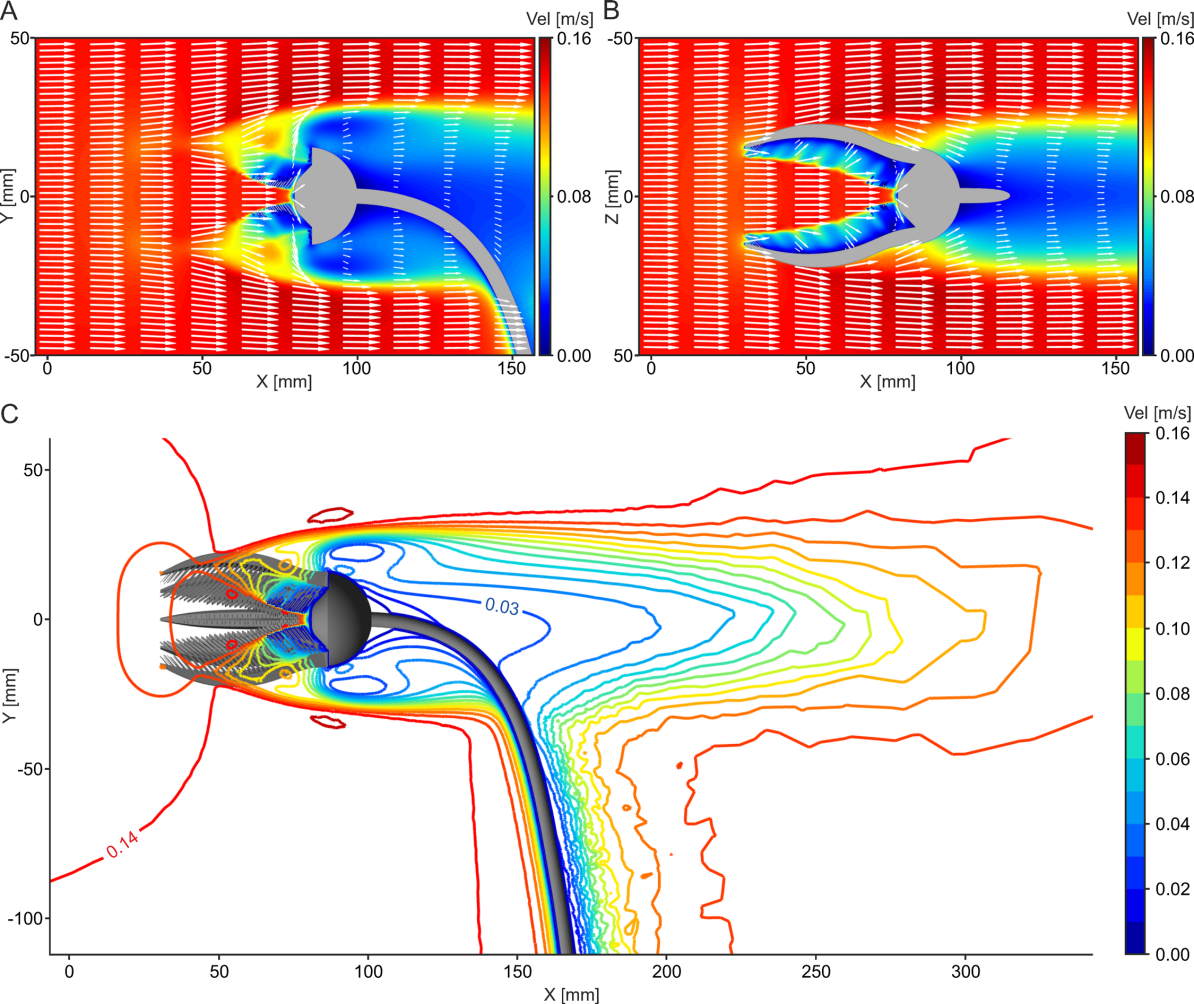
Flow results for the base model and oral inflow at V_init_ = 0.14 m/s. (A) Combined contour-vector plot illustrating flow pattern on XY plane in side view. (B) Combined contour-vector plot illustrating flow pattern on ZX plane in top view. (C) Contourline plot showing velocity distribution on XY plane in side view.

This setting corresponds to Fig 4D. Flow patterns resulting when flow comes from an oral direction are similar for all analyzed models and inflow velocities. The flow reaches the filtering surface of the crown with almost unchanged velocity and the vectors show only slight divergence from the inflow (Fig 11A and 11B). By approaching the pinnules, the water velocity decreases and the vectors show diverging flow around the filtering structures. In this position, the crinoid creates a broad wake in which the water has considerably lower flow velocities (Fig 11C). For all models and velocities, no recirculation zone develops. With increasing distance from the model, the free stream velocity is approached slowly. For V_init_ = 0.14 m/s, the flow velocity has reached 80% of V_init_ in a distance of 200 mm behind the calyx (X = 300 mm) for the base model as well as the one with pinnules spread, 73% with arms opened, and 82% with parts of 3 arms capped.

**Fig 12:**
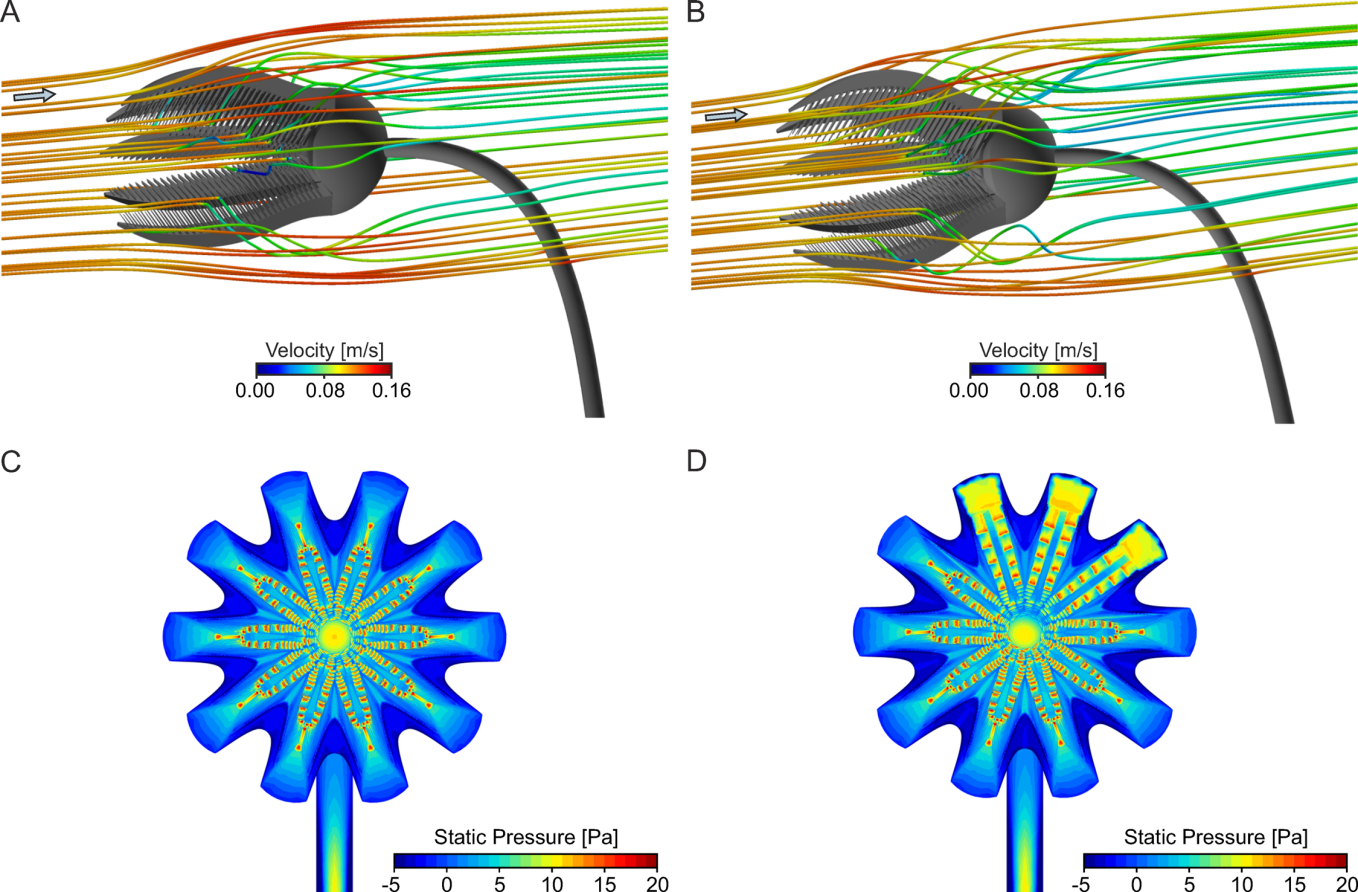
Results of particle tracking simulations and pressure distribution for oral inflow at V_init_ = 0.14 m/s. (A) Pathline plot illustrating straight flow of plankton particles (diameter = 150 μm) through the filter apparatus for the base model. (B) Pathline plot illustrating straight flow of plankton particles (diameter = 150 μm) through the filter apparatus for the model with arms opened. (C) Contour plot illustrating pressure distribution on the oral surface of the base model. (D) Contour plot illustrating pressure distribution on the oral surface of the model with 3 arms capped.

The particle trajectories show that the particles reach the inside of the crown almost without any decrease in velocity, then are slowed down rather abruptly at the filtering surface and are directed through the gaps between the pinnules and arms (Fig 12A, S4 Video) with considerably lower velocities compared to the inflow. Some particles pass the filtering structures two times on their way through the crown. An opening of the arms increases the catchment area that is facing the flow and thus the number of particles that are reaching the oral surface (Fig 12B). The same effect, only to a lesser extent, is achieved by spreading of the pinnules, while loss of parts of the arms results in a reduced catchment area.

In this position, the oral surface faces the flow directly and thus the filtering structures are exposed to the pressure generated by the moving water. The contour plots of the static pressure p_stat_, given in relation to the ambient pressure (Fig 12C and 12D) show that the highest pressure peaks occur on the finest structures, the tips of the arms and pinnules. In addition, high values can be observed on the centre of the calyx, where the mouth is located. For the base model and an inflow velocity of V_init_ = 0.14 m/s, the highest pressure peaks occur on the tips of the pinnules with p_stat_ = 25.64 Pa. While the static pressure distribution changes only slightly with spreading of the pinnules or opening of the arms, loss of parts of arms leads to an irregular pattern with enlarged surfaces facing the current directly, where the arms are capped (Fig 12D). In lower flow velocities, the pressure acting on the oral surface of the crinoid decreases accordingly, while it rises considerably with inflow velocities of V_init_ = 0.50 m/s, reaching peak values of p_stat_ = 253 Pa in relation to the ambient pressure at the tips of the pinnules. Drag in this position (S1 Table) is twice as high compared to the aboral orientation.

### Lateral inflow

**Fig 13:**
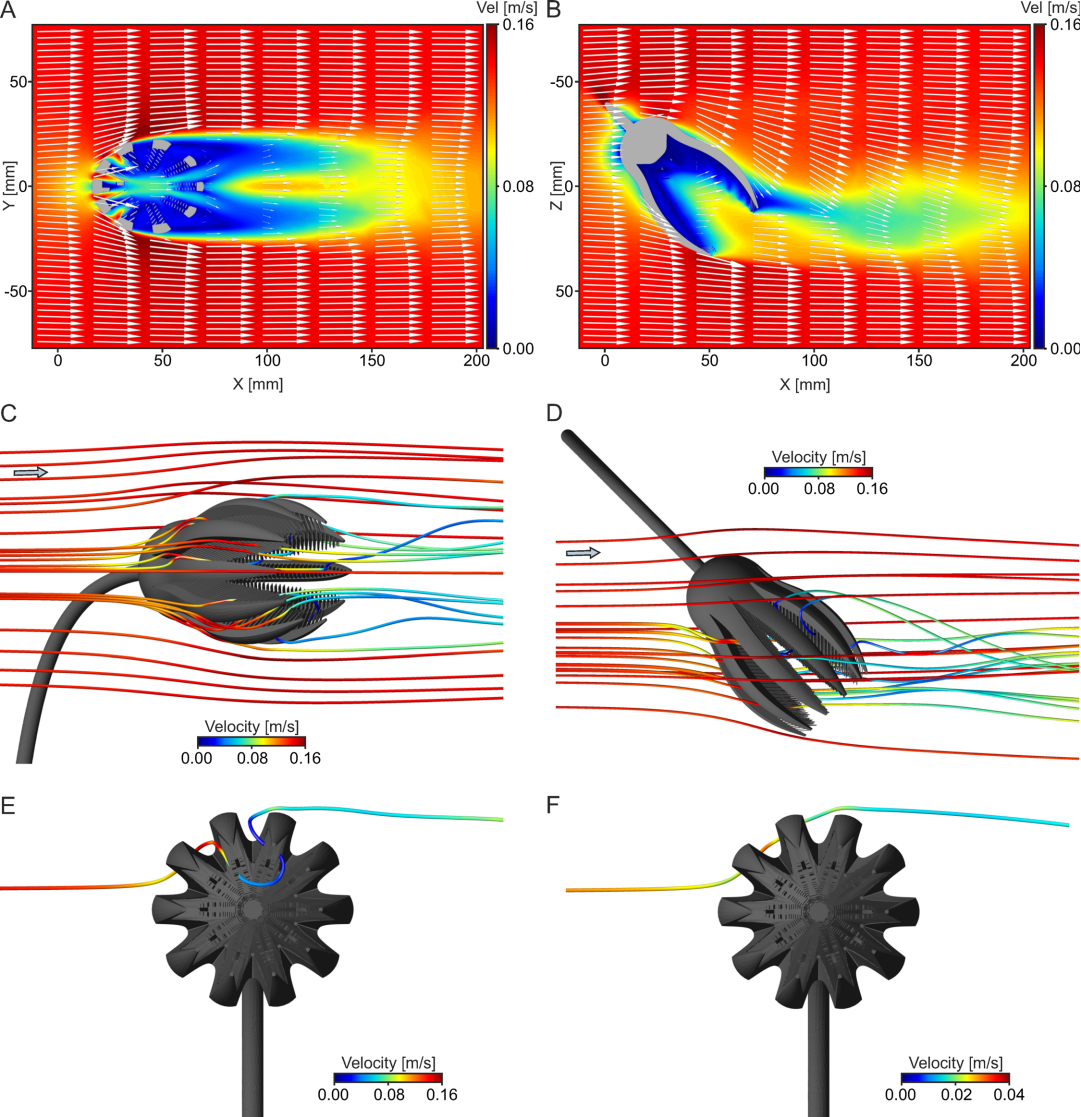
Results for the base model for lateral inflow. (A) Combined contour-vector plot illustrating flow pattern on XY plane in side view at V_init_ = 0.14 m/s. (B) Combined contour-vector plot illustrating flow pattern on ZX plane in top view at V_init_ = 0.14 m/s. (C) Pathline plot illustrating straight flow of plankton particles (diameter = 150 μm) through the filter apparatus in side view at V_init_ = 0.14 m/s. (D) Pathline plot illustrating straight flow of plankton particles (diameter = 150 μm) through the filter apparatus in top view at V_init_ = 0.14 m/s. (E) Pathline of one particle (diameter = 150 μm) that passes the filtering structures 2 times at V_init_ = 0.14 m/s in front view. (F) Pathline of the same particle shown in (E)) at V_init_ = 0.03 m/s in front view.

This setting corresponds to Fig 4E. A lateral inflow results in a relatively straight water movement through the crown (Fig 13A and 13B). Also in this setting, no large scale recirculation zones develop for all models and velocities. The arms and pinnules facing the flow are exposed to high velocity gradients, where the water flows through the gap between the arms with increased velocity. The rest of the filter apparatus is characterized by low flow velocities. Inside the crown, the flow is deviated slightly and the vectors indicate water movement directed towards the pinnules. Behind the crown, an irregular wake forms that has a more limited extent compared to the other two inflow directions.

Particles move through the pinnules facing the flow with decreased velocities, thus approaching the filtering surface directly (Fig 13C and 13D). Some of these perform a loop inside the crown thus passing the filtering structures a second time (Fig 13E). The comparison of the different analyzed inflow velocities reveals that some of the particles that enter the crown at V_init_ = 0.14 m/s, follow a different path at V_init_ = 0.03 m/s and instead are deviated around the crown. Fig 13F exemplary illustrates this different flow behaviour for the same particle shown in Fig 13E. In addition, the particles do not perform loops inside the crown at V_init_ = 0.03 m/s. An increase in velocity to V_init_ = 0.50 m/s hardly changes the flow behaviour of plankton compared to V_init_ = 0.14 m/s, but reduces the duration time of the particles inside the crown.

On the surface facing the flow, three static pressure peaks develop: for V_init_ = 0.14 m/s, the highest values occur on the stalk (p_stat_ = 12.64 Pa), followed by the calyx (p_stat_ = 11.15 Pa) and the proximal parts of the arms (p_stat_ = 8.75 Pa). On the downstream side of the crinoid, the static pressure is generally negative and reaches minimum values of p_stat_ = -8.73 Pa at the calyx. The pressure difference between the upstream and downstream side of the crown is thus relatively high, reaching maximum values of p_stat_ = 19.67 Pa at the calyx (X = 13 mm). For V_init_ = 0.03 m/s, this difference equals p_stat_ = 0.92 Pa, and for V_init_ = 0.50 m/s p_stat_ = 257 Pa.

## Discussion

Crinoid feeding has generated high interest during the last decades (e.g. [4, 11, 13, 14, 52–55]), and the knowledge derived from typical living forms was used for interpretation of their fossil representatives (e.g. [21–27]). These studies, however, were related to stalked crinoids that possibly used the parabolic filtration fan for feeding, while investigations of forms that differ in their morphology and thus may have used a different feeding position are scarce (e.g. [28]). Examples from the Recent are represented by the Holopodidae, which cement directly to the substratum, and have ten stout arms which are not recurved into the water current, but have a funnel-like appearance [56]. The species *Holopus rangii* was hypothesized to be an active raptorial feeder catching benthonic plankton and preferring habitats with only moderate unidirectional flow [57]. The cyrtocrinid genus *Cyathidium*, which is described as part of a "living-fossil community" [58], was suggested to be both a suspension as well as a raptorial feeder [59]. Due to the lack of observations, however, there has been no direct evidence for the feeding behaviour of these crinoids.

It has been suggested that *E*. *liliiformis* was a specialized feeder, being able to obtain nutrients not only by passive, but also by active suspension feeding [30], a feeding strategy that is not known from living crinoids. This conclusion was based on the occurrence of distinctive grooves on the pinnules forming channels with diameters of up to 50 μm, which were reconstructed as being ciliated and thus enabled the crinoid to produce an active feeding current, at least in the innermost part of the crown, close to the mouth. Following the suggestions of [30], active filter feeding could have been used in the proximal parts, and concurrent passive suspension feeding in the distal parts of the arms. A rigorous analysis of flow patterns, however, was not provided.

The presented results show the development of a recirculation current for the aboral inflow direction, behind the crown of *E*. *liliiformis*, which is strong enough to transport plankton particles back into the crown onto the oral surface. Inside the crown, the flow was decelerated, resulting in an increased residence time of food items and thus enhanced capture probability [60, 61]. The deceleration of particles inside the crown enabled capture by the extended tube feet even in high free-stream velocities, because the drag forces acting on the decelerated particles were kept low, probably not exceeding the adhesive forces of mucus which is described for high-velocity environments [35]. When arms were not tightly closed, there was a constant exchange of fluid between the outside and the inside of the crown, promoted by turbulences in the moving water, preventing the formation of stationary circulation and thus the depletion of plankton within the crown. Flow leaving the crown by passing between pinnules, and therefore occurring in a regime of lower local Reynolds number when viscous forces become relevant, may also be related to viscous entrainment [62]. This phenomenon sets in when a small space limited by two orifices extends between regions of higher (in this case flow along the crown exterior) and lower flow velocity (in this case the crown interior). Water will then be drawn out of the crown due to the higher shear stress at its outer surface which may also support particle capture.

Increasing inflow velocities resulted in higher velocity gradients inside the crown and higher velocities of backwards oriented flow, allowing for particles to be transported deeper into the crown and closer to the mouth, an area with dense pinnule spacing. Increasing the opening angle of the arms increases the recirculation zone and therefore the rate of backwards transported particles, as shown by particle tracking animations. These particles reached higher recirculation velocities and performed a larger number of turns inside the crown thus passing the filtering surface several times and increasing the probability to be captured [8]. Changing the opening angle thus probably represented an opportunity by which the crinoid could affect the particle capture success under changing current conditions. The region in front of the crinoid, which particles had to enter to be caught in the recirculation current, however, is relatively confined and only those particles could be caught which passed the crinoid crown quite closely. Items that were passing directly between the arms probably represent an additional food source and recirculation was thus not the only feeding method involved when this posture was attained. Thus, even if spreading of the pinnules only had a small effect on the overall flow pattern, the area which could be available for direct capture in between the arms increased, while the velocity of reversed flow decreased slightly. Predation or loss of parts of the arms due to autotomy, at least if parts of three arms were missing as simulated in the presented study, did not prevent the formation of the recirculation zone, but it changed the particle behaviour considerably. While plankton was still transported back to the crinoid, most of it left the inside of the crown through the gap caused by the missing parts without reaching the oral surface. Thus it can be concluded that already comparatively limited damage of the filtering apparatus resulted in a substantially reduced feeding success.

The analyzed posture of *E*. *liliiformis* with flow coming from the aboral side resembles the survival posture of present-day forms in high or turbulent currents [21, 22]. This tear shape reduces drag forces on the animal, but results in the cessation of feeding due to the closure of the crown. The pressure distributions on the surface of *E*. *liliiformis* for an aboral as well as a lateral inflow show that the highest pressure values occurred on the stalk and the calyx. Drag is a function of pressure difference acting upon the object in front of and behind the body [63], and recirculation behind the crown might have increased the drag acting on the animal. The turbulence model applied in this study, however, did not allow for the derivation of precise drag values.

For the oral inflow direction with the filtering surface facing the flow, particles probably could be captured directly and the size of the filtering surface was correlated to the spreading angle of the pinnules and the opening angle of the arms. With an oral inflow, however, a higher drag resulted such that the animal probably had to compromise by adjusting the opening angle of the crown according to flow velocity. According to [64], upstream capture of plankton is rare in living crinoids and a constant downstream orientation of the filtering surface should be maintained. It was suggested by [5] that this aboral orientation prevents direct impingement of the current on the food grooves and the particle manipulating tube feet. Thus being positioned in a region of low velocities, the risk of losing particles once they were captured would be minimized and retention of the particles facilitated. For fossil stalked crinoids, [64] stated four possibilities of maintaining the downstream orientation of the feeding structures: 1) they were able to swivel their pinnules, as was observed in living comatulids [5], 2) they maintained a constant position, and feeding occurred only during one half-cycle of periodic current reversals, 3) they were able to rotate the whole filter or the arms by an unknown mechanism, or 4) they did not live in areas characterized by bi-directional flow. Due to the different morphology and thus construction of the filter apparatus of *E*. *liliiformis*, the tube feet and food grooves were not exposed to direct current with an oral inflow. The CFD simulations showed that the particles entering the filter apparatus by oral inflow were slowed down considerably between the pinnules thus possibly enabling upstream capture of plankton.

Also lateral inflow, in the presented study exemplary simulated by a rotation of the crown by 45°, may have allowed for feeding, leading to direct flow of particles through the pinnules. In this orientation, no recirculation occurred, but the number of particles passing the filtering surface directly was considerably higher compared to an aboral inflow direction. All feeding postures of *E*. *liliiformis*, including orientation with an aboral inflow, may thus have offered the ability to perform filter feeding under conditions of a dynamically changing flow regime. When flow velocities reached a critical value, the crown may have closed completely.

The results for particle behaviour within the recirculation suggest that particle selection towards smaller plankton diameters (less than 150 μm) existed for *E*. *liliiformis*, which coincides with observations on recent crinoids [10]. Generally, the particle size a crinoid filters and digests is assumed to be related to food groove width and mostly particles smaller than this value will actually be used as food source. Our own observations of the feather star *Antedon bifida* as well as reports of several other researchers [65, 66] showed, however, that also particles well exceeding the width of the food groove in diameter could be handled and were transported into the mouth.

Plankton was likely to be captured by the tube feet, but particle capture rates are difficult to conclude from the flow patterns obtained in this study. For the analyzed scenarios, the Reynolds number of flow around the crown (using crown length as characteristic length) amount to Re = 1,840 for V_init_ = 0.03 m/s, Re = 8,600 for V_init_ = 0.14 m/s, and Re = 30,730 for V_init_ = 0.5 m/s, thus indicating a turbulent flow regime for all model variations. For particle capture, however, local Reynolds numbers directly at the filtering organs, in the vicinity of the boundary layer, are determining the plankton behaviour. Instead of low Reynolds number flow, where viscosity is the dominant force [62], the filter apparatuses of many suspension feeders operate in intermediate Reynolds numbers between 0.1 and 50 with almost balanced inertial and viscous forces [36].

The dimensionless indices for direct interception (NR) and inertial impaction (NI) [12] can be calculated assuming that the primary tube feet of *E*. *liliiformis* had a similar size as those reported for living comatulids [4], e.g. with an average diameter of 50 μm. Based on the local flow velocities at the pinnules, derived from the presented computer simulations, the indices show NR > NI for all analyzed inflow velocities, inflow directions and particle sizes thus indicating an intermediate Reynolds regime. The process of particle capture thus requires an approach that fully models the boundary layer region which, however, is beyond the capability of the k-epsilon approach here used. This model is well suited for high-Reynolds flow and turbulence but does not allow for analysing the realm of low Reynolds processes.

A rough estimation of the rate of particles transported into the crown by backwards oriented flow can be provided as follows. Using that part of the total inlet flow plane which actually interacts with the crown (here the particle injection area with a radius of 25 mm), and the initial velocity of 0.14 m/s, a volume flow rate of about 275 ml/s can be determined. Marine particle concentrations are very variable, but 1000 particles per 100 ml may be taken as a common value in many marine settings [67]. This results in a particle flow rate of about 2.7 10^5^ particles per second and if only 1% of these particles are redirected into the filter, then a total of about 2,750 particles per second are transported backwards into the crown, and about 1.0 10^7^ particles per hour. For an aboral inflow, the simulations show a recirculation of 1–5% particles, for lateral and oral inflow the values are considerably higher and close to 100% may reach the filter. This rough estimation, however, does not allow for the reconstruction of particle capture rates. For studying particle capture inside the crown future work is necessary that addresses the flow and particle behaviour directly at the filter elements, with the macroscopic flow serving as boundary conditions. This would allow for conclusions regarding particle capture effectiveness and thus address the question, if the analysed orientations would suffice to catch enough particles to satisfy the nutritional needs of *E*. *liliiformis*.

The ability to continue feeding with different postures, however, appears to be advantageous in varying flow regimes over being dependent on a specific flow direction. The Middle Triassic Muschelkalk has been reconstructed as a dynamic shallow water environment [38, 44–47], suggesting that frequently changing flow directions occurred. In the subtidal habitat that was populated by *E*. *liliiformis*, these changes did not happen as rapidly and abruptly as in a wave-dominated environment, but flow reversals probably regularly occurred. The lack of muscular articulations in the crinoid stalk precluded an active reorientation of the complete crown, and a postural change, as was observed for recent crinoids by adjusting the arm position [20], might have been achieved in the oral inflow position as well, leading to a passive reorientation. Assuming that fossil crinoids also possessed mutable collagenous tissue [68], they also were able to hold their feeding position by stiffening this specialized tissue, without expending any more energy [9]. Keeping the MCT in a stiff state would have prevented a passive reorientation (similar to a weathervane), and aided in withstanding torques produced by changing flow directions at least in low flow velocities. When velocities reached a critical value, however, destiffening of MCT would have enabled a passive reorientation similar to a “weather vane”, changing from the oral into an aboral posture.

As the hydrodynamic results demonstrate, plankton particles were likely to be able to enter the filtering apparatus by being passively transported with the flowing water, without the necessity of producing an active feeding current. Thus it is concluded that passive suspension feeding was probably the main strategy used by *E*. *liliiformis*, also because it is energetically more favourable [69]. Active filter feeding, however, could have occurred in slack water conditions, when flow velocities were too low for passive suspension feeding (as the presented results indicate, recirculation of plankton stopped at inflow velocities of 0.01 m/s). This behaviour has been reported for different suspension feeding animals (e.g. [70–72]) with the explanation that active filter feeding is more advantageous in low flow velocities, whereas passive filter feeding only works effectively in higher flow velocities. The CFD analyses of *E*. *liliiformis* performed within this study, with varying postures as well as flow conditions, indicate that the filter apparatus of this fossil taxon may have shown a broad functionality in frequently changing flow directions and velocities.

## Supporting Information

S1 Fig Morphology of a typical recent stalked crinoid. Schematic drawing illustrating general morphologic features as well as the typical feeding position, the parabolic filtration fan, where the arms are bent backwards into the flow and the oral surface of the calyx faces downstream.
(TIF)

S2 Fig Result comparison of PIV and CFD at V_init_ = 0.14 m/s, illustrated as linegraph plots of velocity components V and U at 4 different transect lines (locations indicated by dotted lines in Fig 6). A) Line at widest diameter of the calyx; B) Line at the widest diameter of the crown; C) Line directly behind the end of the arms; D) Line in the wake of the crown, 20 measurement points behind the end of the arms. The direct comparison of PIV and CFD results reveals slight differences, especially at the widest diameter of the calyx and arms, while the curve progressions in the wake of the model are almost similar. The deviations can be attributed to irregularities in the handmade model, which is not perfectly symmetrical compared to the computer generated geometry, and can be seen in the asymmetric curve progression. In addition, due to the experimental setup of PIV, some areas were not accessible to the laser so that no results are available from the inside of the crown, while CFD provides complete values. The general flow pattern, however, as well as the recirculation behind the crown, is similar for both methods and thus the experimental data validate the computationally derived flow patterns.
(TIF)

S3 Fig Comparison of the extent of the recirculation zone at V_init_ = 0.14 m/s and V_init_ = 0.50 m/s, displaying velocity component u along line X (cf. Fig 7 and Fig 8) for the base model and arms opened. As the linegraph plots illustrate, the enlargement of the recirculation area (indicated by negative values of velocity component u) is only related to an opening of the arms, but not to an increase in velocity.
(TIF)

S1 Table Drag values in Newton derived from CFD simulations. Even though no experimental drag data exist for validation, the results indicate general trends that seem reasonable with a drag twice as high for the oral compared to the aboral orientation and generally increasing drag with increasing velocity. The absolute values, however, should be interpreted carefully due to limitations of the applied k-epsilon turbulence model.
(XLS)

S2 Table Datasets providing values for Fig 6C and 6D, Fig 7E, Fig 8A and 8B, Fig 9C, S2 Fig and S3 Fig. (XLS)

S1 Video Particle tracking animation illustrating recirculation of plankton for the base model with aboral inflow at V_init_ = 0.14 m/s (Video speed does not reflect real particle velocity). (MP4)

S2 Video Particle tracking animation illustrating particle paths inside the crown for the base model with aboral inflow at V_init_ = 0.14 m/s (Video speed does not reflect real particle velocity). (MP4)

S3 Video Particle tracking animation illustrating particle paths inside the crown for the model with parts of 3 arms capped with aboral inflow at V_init_ = 0.14 m/s (Video speed does not reflect real particle velocity). (MP4)

S4 Video Particle tracking animation illustrating particle paths inside the crown for the base model with oral inflow at V_init_ = 0.14 m/s (Video speed does not reflect real particle velocity). (MP4)

## References

[1] Moore RC, Teichert C. Introduction In: Moore RC, Teichert C, editors. Treatise on Invertebrate Paleontology, Part T Echinodermata 2 Crinoidea. Boulder, Colorado: Geological Society of America and University of Kansas; 1978 pp. T7–T9.

[2] Pawson DL. Phylum Echinodermata. Zootaxa. 2007; 1668:749–764.

[3] Breimer A. Recent crinoids In: Moore RC, Teichert C, editors. Treatise on Invertebrate Paleontology, Part T Echinodermata 2 Crinoidea. Boulder, Colorado: Geological Society of America and University of Kansas; 1978 pp. 9–57.

[4] Meyer DL. Length and spacing of the tube feet in crinoids (Echinodermata) and their role in suspension-feeding. Mar Biol. 1979; 51:361–369.

[5] Meyer DL. Food and Feeding Mechanisms: Crinozoa In: Jangoux M, Lawrence JM, editors. Echinoderm Nutrition. Rotterdam: A. A. Balkema; 1982 pp. 25–42.

[6] Lawrence J. Acquisition of Nutrients In: Calow P, editor. A Functional Biology of Echinoderms. London, Sidney: Croom Helm Ltd. Publishers; 1987 pp. 17–31.

[7] Heinzeller T, Welsch U. Crinoidea. Microscopic Anatomy of Invertebrates. 1994; 14:9–148.

[8] Warner GF. On the shape of passive suspension feeders In: Keegan BF, Ceideigh PO, Boaden PJS, editors. Biology of Benthic Organisms. Oxford: Pergamon; 1977 pp. 567–576.

[9] Baumiller TK. Crinoid Ecological Morphology. Annu Rev Earth Planet Sci. 2008; 36:221–249.

[10] Featherstone CM, Messing CG, McClintock JB. Dietary composition of two bathyal stalked crinoids: *Neocrinus decorus* and *Endoxocrinus parrae* (Echinodermata: Crinoidea: Isocrinidae). In: Mooi R, Telford M, editors. Ninth international Echinoderm Conference. San Francisco, California, USA: Balkema; 1998. pp. 155–160.

[11] Kitazawa K, Oji T, Sunamura M. Food composition of crinoids (Crinoidea: Echinodermata) in relation to stalk length and fan density: their paleoecological implications. Mar Biol. 2007; 152(4):959–968.

[12] Baumiller TK, Messing CG. Stalked Crinoid Locomotion, and its Ecological and Evolutionary Implications. Palaeontol Electronica. 2007; 1(10):10.1.2A.

[13] Macurda DB, Meyer DL. Feeding Posture of Modern Stalked Crinoids. Nature. 1974; 247:394–396.

[14] Baumiller TK. Effects of filter porosity and shape on fluid flux: Implications for the biology and the evolutionary history of stalked crinoids. Echinoderm Biology: Proceedings of the 6th International Echinoderm Conference, Victoria 1987. 1988:786.

[15] Conan G, Roux M, Sibuet M. A photographic survey of a population of the stalked crinoid *Diplocrinus (Annacrinus) wyvillethomsoni* (Echinodermata) from the bathyal slope of the Bay of Biscay. Deep Sea Res A. 1981; 28A(5):441–453.

[16] Jørgensen CB. Fluid mechanical aspects of suspension feeding. Mar Ecol Prog Ser. 1983; 11:89–103.

[17] Leonard AB. Functional response in *Antedon mediterranea* (Lamarck) (Echinodermata: Crinoidea): the interaction of prey concentration and current velocity on a passive suspension-feeder. J Exp Mar Biol Ecol. 1989; 127(1):81–103.

[18] Baumiller TK. Crinoid stalks as cantilever beams and the nature of stalk ligament. Neues Jahrb Geol Palaontol Abh. 1993; 190(2/3):279–297.

[19] Messing CG. Submersible observations of deep-water crinoid assemblages in the tropical western Atlantic Ocean. Proceedings of the 5th International Echinoderm Conference in Galway. 1985:185–193.

[20] Baumiller TK, LaBarbera M, Woodley JD. Ecology and Functional Morphology of the Isocrinid *Cenocrinus asterius* (Linnaeus) (Echinodermata: Crinoidea): In situ and Laboratory Experiments and Observations. Bull Mar Sci. 1991; 48(3):731–748.

[21] Haugh BN. Biodynamic and phyletic paradigms for sensory organs in camerate crinoids. Lethaia. 1978; 11:145–173.

[22] Roux M. Evolutionary ecology and biogeography of recent stalked crinoids as a model for the fossil record. Echinoderm Studies. 1987; 2:1–53.

[23] Welch JR. Flume study of simulated feeding and hydrodynamics of a Paleozoic stalked crinoid. Paleobiology. 1978; 4:89–95.

[24] Baumiller TK, Plotnick RE. Rotational stability in stalked crinoids and the function of wing plates in *Pterotocrinus depressus*. Lethaia. 1989; 22:317–326.

[25] Ausich W. Palaeoecology and history of the Calceocrinidae (Palaeozoic Crinoidea). Palaeontology. 1986; 29(1):85–99.

[26] Brower JC. The application of filtration theory to food gathering in Ordovician crinoids. J Paleontol. 2007; 81(6):1284–1300.

[27] Brower JC. Paleoecology of suspension-feeding echinoderm assemblages from the Upper Ordovician (Katian, Shermanian) Walcott-Rust Quarry of New York. J Paleontol. 2011; 85(2):369–391.

[28] Bohatý J. Revision of the flexible crinoid genus *Ammonicrinus* and a new hypothesis on its life mode. Acta Palaeontol Pol. 2011; 56(3):615–639.

[29] Ausich WI, Brett CE, Hess H, Simms MJ. Crinoid form and function In: Hess H, Ausich WI, Brett CE, Simms MJ, editors. Fossil Crinoids. Cambridge: Cambridge University Press; 1999 pp. 3–30.

[30] Jefferies RPS. The arm structure and mode of feeding of the Triassic crinoid *Encrinus liliiformis*. Palaeontology. 1989; 32(3):483–497.

[31] Rubenstein DI, Koehl MAR. The Mechanisms of filter feeding: some theoretical considerations. Am Nat. 1977; 111(981):981–994.

[32] Silvester NR. Some Hydrodynamic Aspects of Filter Feeding with Rectangular-Mesh Nets. J Theor Biol. 1983; 103:265–286.

[33] LaBarbera M. Feeding Currents and Particle Capture Mechanisms in Suspension Feeding Animals. Am Zool. 1984; 24:71–84.

[34] Patterson MR. The Effects of Flow on Polyp-Level Prey Capture in an Octocoral, *Alcyonium siderium*. Biol Bull. 1991; 180:93–102. 10.2307/1542432 29303631

[35] Shimeta J, Jumars PA. Physical mechanisms and rates of particle capture by suspension-feeders. Oceanography and Marine Biology: An Annual Review. 1991; 29:191–257.

[36] Humphries S. Filter feeders and plankton increase particle encounter rates through flow regime control. Proc Natl Acad Sci U S A. 2009; 106(19):7882–7887. doi: 10.1073/pnas.0809063106 19416879 10.1073/pnas.0809063106 PMC2683099

[37] Riisgård HU, Larsen PS. Particle capture mechanisms in suspension-feeding invertebrates. Mar Ecol Prog Ser. 2010; 418:255–293.

[38] Hagdorn H. Triassic Muschelkalk of Central Europe In: Hess H, Ausich WI, Brett CE, Simms MJ, editors. Fossil Crinoids. Cambridge: Cambridge University Press; 1999 pp. 164–176.

[39] Linck O. Die Muschelkalk-Seelilie *Encrinus liliiformis*—Ergebnisse einer Grabung. Naturwissenschaftliche Monatsschrift, Aus der Heimat 1954; 62:225–235.

[40] Hess H. Encrinida In: Seldon PA, editor. Treatise on Invertebrate Paleontology Part T Echinodermata 2 Revised Crinoidea. Lawrence, Kansas: The University of Kansas Paleontological Institute; 2011 pp. 28–41.

[41] Birenheide R, Motokawa T. Contractile connective tissue in crinoids. Biol Bull. 1996; 191:1–4. 8776839 10.2307/1543055

[42] Feist-Burkhardt S, Götz AE, Szulc J, Borkhataria R, Geluk M, Haas J, et al Triassic In: McCann T, editor. The Geology of Central Europe Volume 2: Mesozoic and Cenozoic. London: The Geological Society; 2008 pp. 749–821.

[43] Geyer OF, Gwinner MP. Geologie von Baden-Württemberg. Stuttgart: Schweizerbart; 2011.

[44] Vollrath A. Zur Entwicklung des Trochitenkalkes zwischen Rheintal und Hohenloher Ebene. Jahreshefte des geologischen Landesamtes Baden-Württemberg. 1957; 2:119–134.

[45] Aigner T. Storm depositional systems. Dynamic stratigraphy in modern and ancient shallow-marine sequences. Lecture Notes in Earth Sciences. 1985; 3(174).

[46] Hagdorn H. The Muschelkalk in Germany—An Introduction In: Hagdorn H, editor. Muschelkalk—A Field Guide. Korb: Goldschneck-Verlag; 1991; 7–21.

[47] Aigner T, Bachmann GH. Sequence-stratigraphic framework of the German Triassic. Sediment Geol. 1992; 80(1–2):115–135.

[48] Linck O. Stratigraphische, stratinomische und ökologische Betrachtungen zu *Encrinus liliiformis* Lamarck. Jh geol Landesamt Baden-Württemberg. 1965; 7:123–148.

[49] Dynowski JF, Nebelsick JH. Ecophenotypic variations of *Encrinus liliiformis* (Echinodermata: Crinoidea) from the middle Triassic Muschelkalk of Southwest Germany. Swiss Journal of Palaeontology. 2011; 130:53–67.

[50] van Ierland ET, Peperzak L. Separation of marine seston and density determination of marine diatoms by density gradient centrifugation. J Plankt Res. 1984; 6/1:29–44.

[51] Dynowski JF. Hydrodynamic analysis of suspension feeding in recent and fossil crinoids. Doctoral Dissertation, Eberhard Karls Universität Tübingen. 2015. Available: http://hdl.handle.net/10900/60347

[52] Meyer DL. Feeding behavior and ecology of shallow-water unstalked crinoids (Echinodermata) in the Caribbean Sea. Mar Biol. 1973; 22(2):105–129.

[53] Holland ND, Leonard AB, Strickler JR. Upstream and Downstream during Suspension Feeding by *Oligometra serripinna* (Echinodermata: Crinoidea) under Surge Conditions. Biol Bull. 1987; 173:552–556. 10.2307/1541700 29320227

[54] Baumiller TK. Importance of hydrodynamic lift to crinoid autecology, or, could crinoids function as kites? J Paleontol. 1992; 66(4):658–665.

[55] Kitazawa K, Oji T. Particle selection by the sea lily *Metacrinus rotundus* Carpenter 1884 (Echinodermata, Crinoidea). J Exp Mar Biol Ecol. 2010; 395:80–84.

[56] Donovan SK, Pawson DL. A new species of the sessile crinoid *Holopus* d'Orbigny from the tropical western Atlantic, with comments on holopodid ecology (Echinodermata: Crinoidea: Holopodidae). Zootaxa. 2008; 1717:31–38.

[57] Grimmer JC, Holland ND. The Structure of a Sessile, Stalkless Crinoid (*Holopus rangii*). Acta Zool. 1990; 71(2):61–67.

[58] Wisshak M, Neumann C, Jakobsen J, Freiwald A. The 'living-fossil community' of the cyrtocrinid *Cyathidium foresti* and the deep-sea oyster *Neopycnodonte zibrovii* (Azores Archipelago). Palaeogeogr Palaeoclimatol Palaeoecol. 2009; 271(1):77–83.

[59] Heinzeller T, Fechter H. Microscopical Anatomy of the Cyrtocrinid *Cyathidium meteorensis (sive foresti)* (Echinodermata, Crinoidea). Acta Zool. 1995; 76(1):25–34.

[60] Lasker HR. A Comparison of the Particulate Feeding Abilities of Three Species of Gorgonian Soft Coral. Mar Ecol Prog Ser. 1981; 5:61–67.

[61] Patterson MR. Patterns of whole colony prey capture in the octocoral, *Alcyonium siderium*. Biol Bull. 1984; 167:613–629. 10.2307/1541414 29320270

[62] Vogel S. Life in Moving Fluids: The Physical Biology of Flow. 2nd, revised and expanded ed. Princeton, New Jersey: Princeton University Press; 1996.

[63] Hucho W-H. Aerodynamik der stumpfen Körper. Wiesbaden: Vieweg+Teubner Verlag; 2011.

[64] Baumiller TK. Crinoid Functional Morphology. Paleontological Society Papers. 1997; 3:45–68.

[65] La Touche RW, West AB. Observations on the Food of *Antedon bifida* (Echinodermata: Crinoidea). Mar Biol. 1980; 60:39–46.

[66] Messing CG. Living Comatulids. Paleontological Society Papers. 1997; 3:3–30.

[67] Best BA. Passive Suspension Feeding in a Sea Pen: Effects of Ambient Flow on Volume Flow Rate and Filtering Efficiency. Biol Bull. 1988; 175:332–342.

[68] Wilkie IC, Candia Carnevali MD, Trotter JA. Mutable collagenous tissue: Recent progress and an evolutionary perspective In: Heinzeller T, Nebelsick JH, editors. Echinoderms: München. London: Taylor & Francis Group; 2004 pp. 371–378.

[69] Gili J-M, Coma R. Benthic suspension feeders: their paramount role in littoral marine food webs. TREE. 1998; 13(8):316–321. 21238320 10.1016/s0169-5347(98)01365-2

[70] Trager GC, Hwang J-S, Strickler JR. Barnacle suspension-feeding in variable flow. Mar Biol. 1990; 105:117–127.

[71] Trager G, Genin A. Flow Velocity Induces a Switch From Active to Passive Suspension Feeding in the Porcelain Crab *Petrolisthes leptocheles* (Heller). Biol Bull. 1993; 185:20–27. 10.2307/1542127 29300601

[72] Wildish D, Kristmanson D. Benthic suspension feeders and flow. Cambridge: Cambridge University Press; 1997.

