# Supplementary figures and images for: Computational Fluid Dynamics Analysis of the Fossil Crinoid Encrinus liliiformis (Echinodermata: Crinoidea)
Source: PLoS One. 2016 May 31;11(5):e0156408. doi: 10.1371/journal.pone.0156408 (PMC4887110; doi:10.1371/journal.pone.0156408)

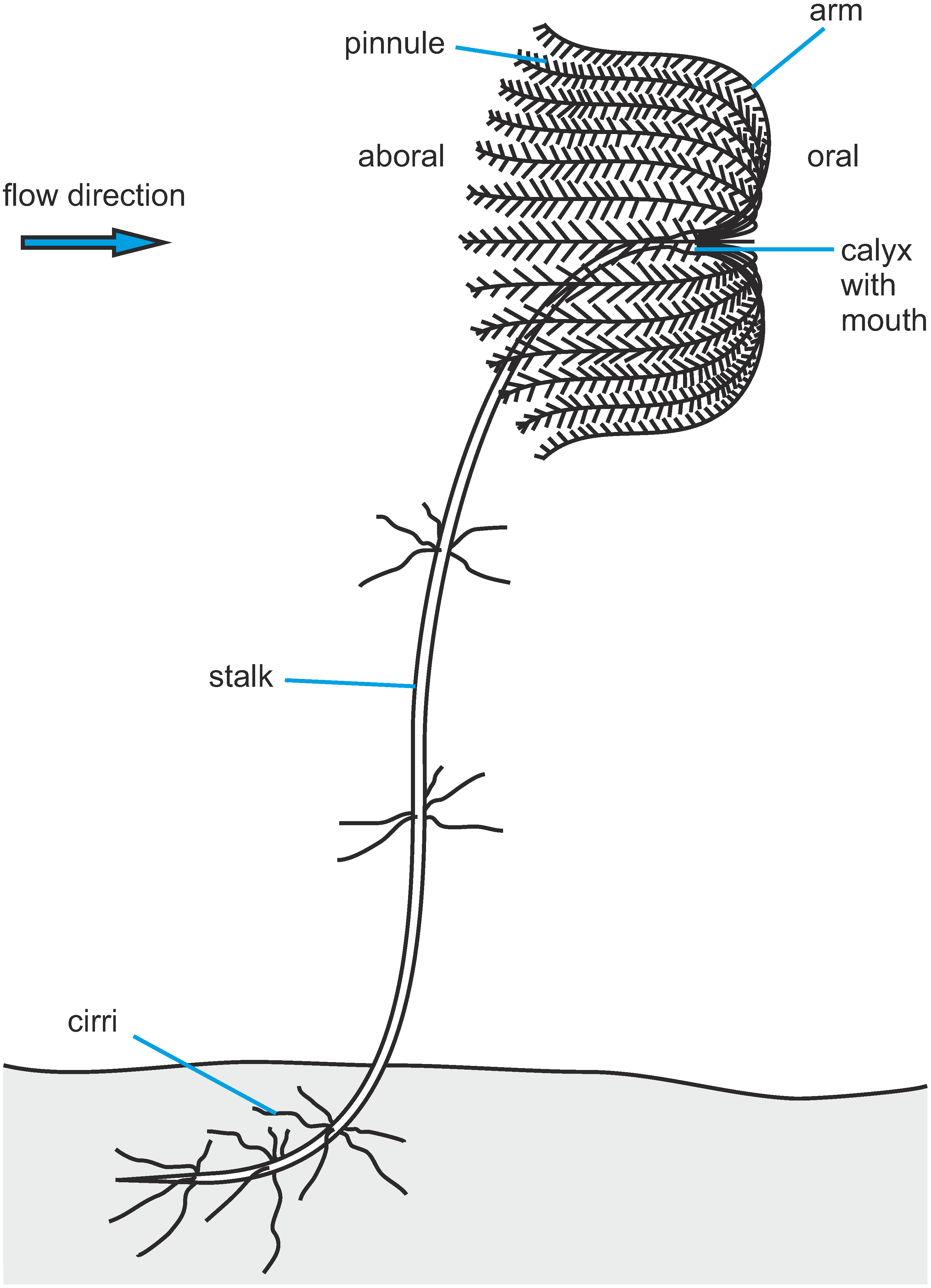

Supplement: S1 Fig — Schematic drawing illustrating general morphologic features as well as the typical feeding position, the parabolic filtration fan, where the arms are bent backwards into the flow and the oral surface of the calyx faces downstream. (TIF) [file pone.0156408.s001.tif]

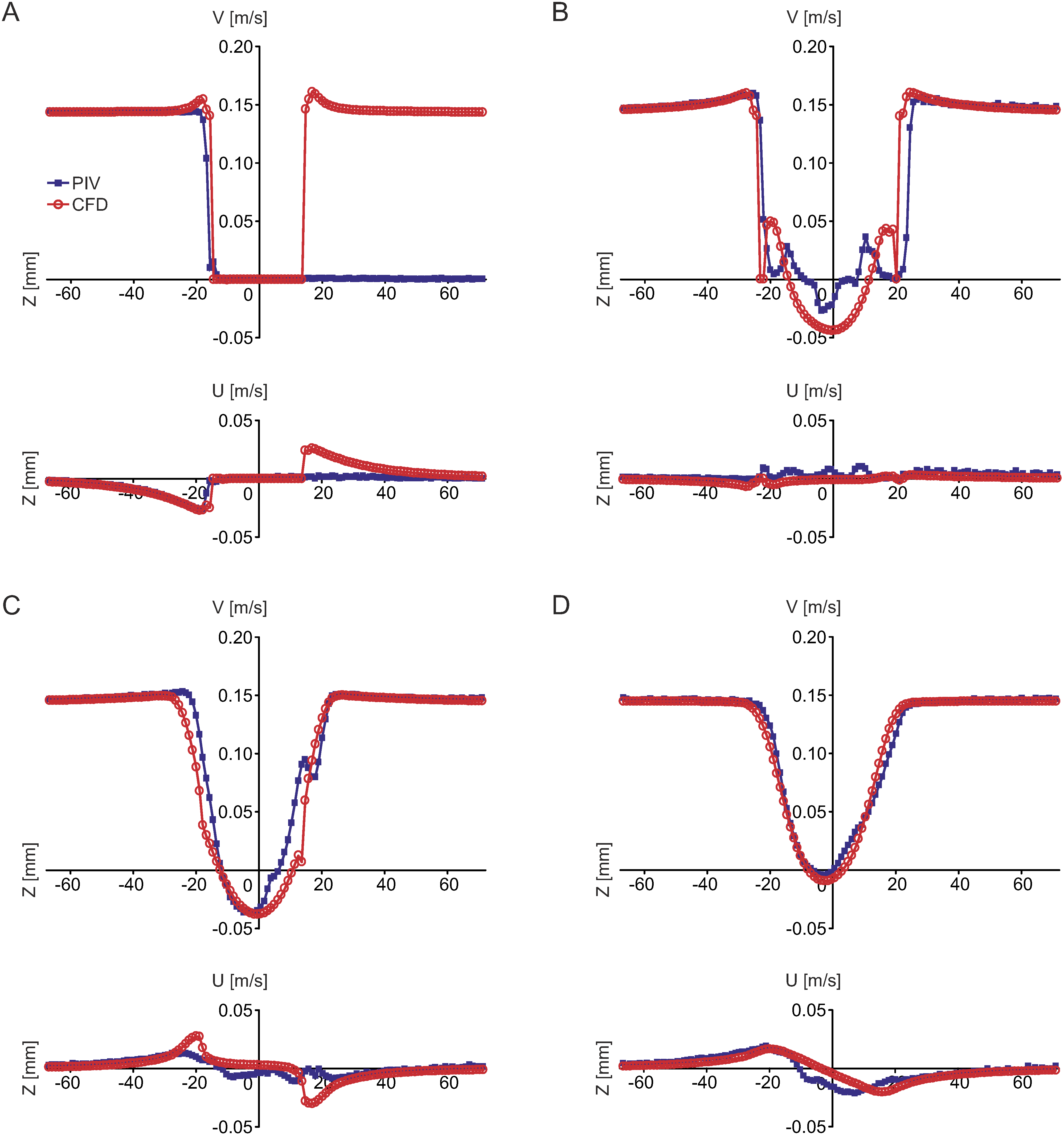

Supplement: S2 Fig — A) Line at widest diameter of the calyx; B) Line at the widest diameter of the crown; C) Line directly behind the end of the arms; D) Line in the wake of the crown, 20 measurement points behind the end of the arms. The direct comparison of PIV and CFD results reveals slight differences, especially at the widest diameter of the calyx and arms, while the curve progressions in the wake of the model are almost similar. The deviations can be attributed to irregularities in the handmade model, which is not perfectly symmetrical compared to the computer generated geometry, and can be seen in the asymmetric curve progression. In addition, due to the experimental setup of PIV, some areas were not accessible to the laser so that no results are available from the inside of the crown, while CFD provides complete values. The general flow pattern, however, as well as the recirculation behind the crown, is similar for both methods and thus the experimental data validate the computationally derived flow patterns. (TIF) [file pone.0156408.s002.tif]

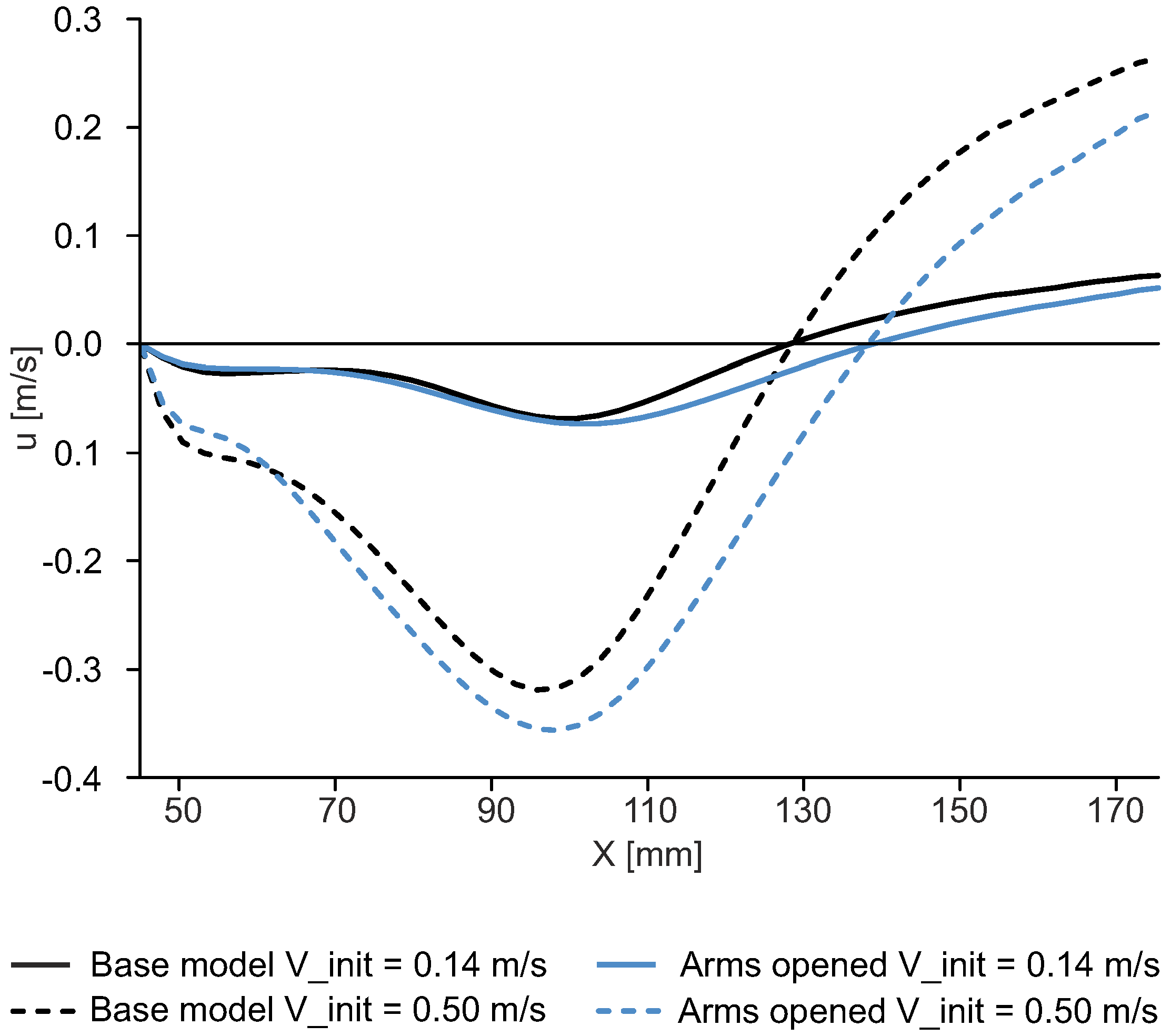

Supplement: S3 Fig — As the linegraph plots illustrate, the enlargement of the recirculation area (indicated by negative values of velocity component u) is only related to an opening of the arms, but not to an increase in velocity. (TIF) [file pone.0156408.s003.tif]
